# Unbiased complexome profiling and global proteomics analysis reveals mitochondrial impairment and potential changes at the intercalated disk in presymptomatic R14^Δ/+^ mice hearts

**DOI:** 10.1371/journal.pone.0311203

**Published:** 2024-10-24

**Authors:** Brian Foo, Hugo Amedei, Surmeet Kaur, Samir Jaawan, Angela Boshnakovska, Tanja Gall, Rudolf A. de Boer, Herman H. W. Silljé, Henning Urlaub, Peter Rehling, Christof Lenz, Stephan E. Lehnart

**Affiliations:** 1 Department of Cardiology and Pneumology, Heart Research Center Göttingen, Cellular Biophysics and Translational Cardiology Section, University Medical Center Göttingen, Göttingen, Germany; 2 Cluster of Excellence “Multiscale Bioimaging: from Molecular Machines to Networks of Excitable Cells” (MBExC), University of Göttingen, Göttingen, Germany; 3 Department of Clinical Chemistry, University Medical Center Göttingen, Göttingen, Germany; 4 Department of Cellular Biochemistry, University Medical Center Göttingen, Göttingen, Germany; 5 Department of Cardiology, University Medical Center Groningen, University of Groningen, Groningen, the Netherlands; 6 Department of Cardiology, Erasmus MC, Thorax Center, Cardiovascular Institute, Rotterdam, the Netherlands; 7 Bioanalytical Mass Spectrometry Group, Max Planck Institute for Multidisciplinary Sciences, Göttingen, Germany; Army Medical University, CHINA

## Abstract

Phospholamban (PLN) is a sarco-endoplasmic reticulum (SER) membrane protein that regulates cardiac contraction/relaxation by reversibly inhibiting the SERCA2a Ca^2+^-reuptake pump. The R14Δ-PLN mutation causes severe cardiomyopathy that is resistant to conventional treatment. Protein complexes and higher-order supercomplexes such as intercalated disk components and Ca^+2^-cycling domains underlie many critical cardiac functions, a subset of which may be disrupted by R14Δ-PLN. Complexome profiling (CP) is a proteomics workflow for systematic analysis of high molecular weight (MW) protein complexes and supercomplexes. We hypothesize that R14Δ-PLN may alter a subset of these assemblies, and apply CP workflows to explore these changes in presymptomatic R14^Δ/+^ mice hearts. Ventricular tissues from presymptomatic 28wk-old WT and R14^Δ/+^ mice were homogenized under non-denaturing conditions, fractionated by size-exclusion chromatography (SEC) with a linear MW-range exceeding 5 MDa, and subjected to quantitative data-independent acquisition mass spectrometry (DIA-MS) analysis. Unfortunately, current workflows for the systematic analysis of CP data proved ill-suited for use in cardiac samples. Most rely upon curated protein complex databases to provide ground-truth for analysis; however, these are derived primarily from cancerous or immortalized cell lines and, consequently, cell-type specific complexes (including cardiac-specific machinery potentially affected in R14Δ-PLN hearts) are poorly covered. We thus developed PERCOM: a novel CP data-analysis strategy that does not rely upon these databases and can, furthermore, be implemented on widely available spreadsheet software. Applying PERCOM to our CP dataset resulted in the identification of 296 proteins with disrupted elution profiles. Hits were significantly enriched for mitochondrial and intercalated disk (ICD) supercomplex components. Changes to mitochondrial supercomplexes were associated with reduced expression of mitochondrial proteins and maximal oxygen consumption rate. The observed alterations to mitochondrial and ICD supercomplexes were replicated in a second cohort of “juvenile” 9wk-old mice. These early-stage changes to key cardiac machinery may contribute to R14Δ-PLN pathogenesis.

## 1. Introduction

Cardiac contraction/relaxation is regulated by controlled cycles of Ca^2+^ release and reuptake from the sarco-endoplasmic reticulum (SER), mediated by the Ryanodine receptor 2 (RyR2) Ca^2+^-release channel and the sarcoplasmic/endoplasmic reticulum (SER) Ca^2+^ ATPase 2a (SERCA2a) reuptake pump, respectively [1]. Phospholamban (PLN) is a 52-aa tail-anchored SER-membrane protein that plays a key role in regulating SERCA2a function [2]. PLN reversibly binds-to and inhibits SERCA2a; this interaction is negatively regulated by phosphorylation of PLN at Ser16 by protein kinase A (PKA, in response to adrenergic signaling) and Thr17 by Ca^2+^/calmodulin-dependent protein kinase II (CaMKII, in response to adrenergic signaling and/or increased cytosolic Ca^2+^) [2]. Several definitively pathogenic disease-associated PLN mutations have been described to-date, including R9C [3], L39stop [4] and R14Δ [5].

The R14Δ-PLN mutation is found worldwide, with a particularly high prevalence the Netherlands and Greece where it is among the leading forms of genetic cardiomyopathy [6]. It is associated with arrhythmogenic and/or dilated cardiomyopathy (ACM/DCM), frequently resulting in heart failure at adolescence and beyond that is resistant to conventional treatment [7, 8]. On the molecular level, R14Δ-PLN is associated with loss of PKA binding, resulting in PLN hypo-phosphorylation leading to altered SERCA2a regulation and Ca^2+^-handling dysfunction [5, 9, 10]. On the cellular level, R14Δ-PLN is associated with a complex phenotype involving accelerated Ca^2+^-handling dynamics, formation of PLN-positive perinuclear SER membrane clusters, upregulated UPR signaling and mitochondrial dysregulation [7, 11–14]. Despite over a decade of work, the mechanisms linking R14Δ-PLN to disease pathology remain poorly understood.

Many proteins assemble into multimeric and/or multi-protein complexes with well-defined molecular composition and function [15, 16]. Notable examples include mitochondrial respiratory-chain complexes I-V [17], SERCA2a dimers [18], Na^+^/K^+^-ATPase dimers [19] and voltage-gated K^+^-channel tetramers [20]. Protein complexes may form further higher-order supercomplexes and subcellular nanodomains, which may be characterized by larger size (≥ 1 MDa), fluid composition and/or stoichiometry, and coordination of several distinct functions such as signaling, cation transport and membrane-tethering [15, 21]. There is a growing appreciation that disruption of these super-assemblies may contribute to cardiac diseases. Examples include disruption of the SER Ca^2+^-handling supercomplex in atrial fibrillation [22], SER-plasma membrane (PM) Ca^2+^-release unit (CRU) in heart failure [23], and intercalated disk components during ischemia-reperfusion injury [24] and ACM [25]. Despite a growing appreciation that assembly of proteins into higher-order complexes and/or supercomplexes influences biological function, exploring their impact in health and disease remains a significant challenge. Current molecular and genetic manipulations (e.g. treatment with receptor agonists or knockdown of specific genes), while well-suited to manipulation of individual proteins or cellular signaling pathways, might only impact protein complexes indirectly and/or incompletely (e.g. ablation of a specific assembly factor or inhibition of a single subunit). Furthermore, while biochemical, biophysical and enzymatic characterization of individual proteins can be done using purified recombinant proteins expressed in *E*. *coli* or yeast [26], similar studies on entire complexes require stringent purification from the native environment. Consequently, the biological function of protein complexes must often be inferred from descriptive data and by correlating protein complex alterations with observed phenotypes. Such was the case when changes to the SER Ca^2+^-handling supercomplex were associated with atrial fibrillation, a common form ACM [22].

**Fig 1:**
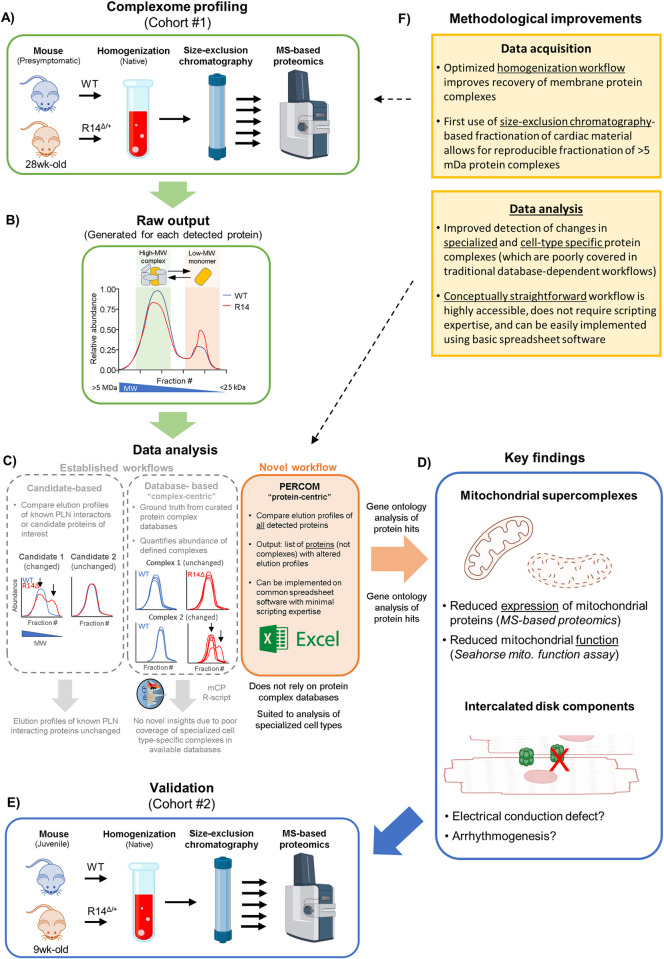
Graphical overview of study. A) Overview of SEC-MS based complexome profiling workflow to identify changes in high-MW protein complexes in R14Δ-PLN mouse hearts. Cardiac tissues from a cohort of 28wk-old ♂ WT and R14^Δ/+^ mice (Cohort #1) were solubilized under non-denaturing conditions and protein complexes fractionated based on apparent molecular weight (MW) by size-exclusion chromatography (SEC). Each fraction was then subject to MS-based proteomics. B) CP generates an “elution profile” of relative abundance vs. elution fraction# for each detected protein. A hypothetical example protein (yellow protein) elutes as two peaks: a low-MW peak corresponding to monomers or low-order assemblies (orange), and a high-MW peak corresponding to higher-order protein complexes or supercomplexes (green). The elution profiles of other proteins comprising this protein complex/supercomplex (grey proteins) are not shown for clarity. Redistribution towards low-MW fractions in R14^Δ/+^ mice may indicate disruption of the high-MW assembly. C) Three modes of data-analysis were tested. Candidate-based strategies (*left*) involve manually comparing elution profiles of known PLN-interacting proteins or other candidates of interest. The illustrated example shows the elution profiles of two hypothetical candidate proteins in WT (blue) and R14^Δ/+^ (red) mice, with candidate 1 (but not candidate 2) being altered in R14^Δ/+^ (arrows). Database-based complex-centric approaches (*middle*) are automated workflows, which compare the abundance of known protein complexes. Curated protein complex databases provide ground-truth for analysis. The illustrated example shows the elution profiles of two hypothetical protein complexes. Each line represents the elution profile of a single subunit comprising the complex. Protein complex ID and subunit composition information is provided by the protein complex database selected for ground-truth. In this example, the abundance of protein complex 2 is reduced in R14^Δ/+^ mice. A major drawback of this approach is the severe underrepresentation and poor coverage of specialized and cell-type specific complexes in these databases, limiting their usefulness in analyzing CP data from specialized tissues e.g. mouse myocardium. PERCOM (*right*) is a novel “protein-centric” strategy that overcomes these limitations by systematically analyzing all detected proteins in an untargeted manner and identifying those with altered elution profiles. Crucially, for our application, it is not constrained by the limited coverage of specialized and cell-type specific complexes in currently available databases. In addition, it is conceptually straightforward, highly accessible, and easily implemented on common spreadsheet software. D) PERCOM was able to identify alterations in 296 proteins (out of 3055 detected). Gene ontology analysis found an enrichment for mitochondrial supercomplex and cardiac-specific intercalated disk (ICD) components among our hits. Notably, conventional candidate-based and complex-centric (via the mCP R-script) data-analysis workflows failed to identify these pathways. Orthogonal techniques revealed decreased mitochondrial protein expression and function in R14^Δ/+^ mice, which may be functionally linked to the complexome-level changes. In parallel, alterations in intercalated disk structures may contribute to the R14Δ-PLN conduction defect and arrhythmogenic phenotype. E) Critically, key changes to mitochondrial and intercalated disk supercomplexes were reproducibly detected in a second cohort (Cohort #2) of juvenile 9wk-old ♂ WT and R14^Δ/+^ mice. F) Key methodological improvements include development of improved homogenization and solubilization workflows with improved protein complex preservation, and use of SEC instead of the BNE fractionation techniques employed in previous cardiac studies. In addition, PERCOM proved more suited to systematic analysis of CP datasets from specialized cell types such as myocardium. Each cohort consisted of a single ♂ WT and a single R14^Δ/+^ mouse (N = 1). All logo copyrights belong to their respective owners.

Complexome Profiling (CP) is a systems-biology approach to study changes in the composition of protein complexes. A basic experimental workflow and example outputs are shown in Fig 1A and 1B. Briefly: biological samples are homogenized under non-denaturing conditions and fractionated based on apparent molecular weight (MW) using techniques such as gradient centrifugation [27, 28], blue-native electrophoresis [29] (BNE) or size-exclusion chromatography (SEC, shown in this example) [30–32]. Each fraction (which ideally correspond to a discrete apparent MW-range) is then analyzed by bottom-up mass spectrometry (MS)-based proteomics (Fig 1A). Raw data output may be formatted as a matrix containing the abundance of each detected protein across each elution fraction, with elution fraction number correlating with MW. Any given protein may be expected to elute at the MW-fraction corresponding to its predicted MW; in the case of PLN, at a predicted MW of ~6 kDa. Should the protein be incorporated into higher-MW protein complexes or supercomplexes, a subpopulation would also elute at higher-MW fractions corresponding to the apparent MW of that complex; in the case of PLN incorporated into the SER Ca^+2^-handling supercomplex, a broad peak at 2–5 MDa was observed via BNE-MS based complexome profiling in previous work [22]. Furthermore, proteins that are part of the same complex/supercomplex would be expected to at-least partially co-elute at these high-MW fractions. Changes in elution profiles between conditions may indicate alterations in protein complex integrity/assembly. This is illustrated in the hypothetical elution profiles shown in Fig 1B. CP datasets may be analyzed using candidate-based approaches (i.e. manually comparing elution profiles of candidate proteins, Fig 1C *left*) or systematic analysis workflows, frequently assisted by software packages or scripts such as mCP [33], Ccprofiler [34, 35] or ComplexBrowser (Fig 1C *middle*) [36]. These scripts typically quantify the abundance of known protein complexes (based on peak analysis of known subunits) and output a list of complexes with altered abundances between experimental conditions. Curated, publicly available protein complex databases such as CORUM [37] or ComplexPortal [38] provide ground-truth for these targeted (complex-centric) strategies.

While these techniques have been used with great success to identify co-migrating/eluting proteins in immortalized cell lines and *in-vitro* systems, allowing for detailed characterization of known protein complexes and *de novo* identification of new complexes [34, 35, 39, 40], several shortcomings in both data acquisition and data analysis steps prevent widespread application of this tool to identify disease-associated changes in specialized tissue types such as the myocardium. Successful data acquisition requires optimized workflows for homogenization and fractionation of complex tissues under native conditions. Furthermore, systematic data analysis strategies frequently rely upon curated protein complex databases for ground truth. Current databases are curated predominantly from immortalized and/or cancer cell lines. As a result, cardiac-specific complexes such as CRUs or ICD components are severely underrepresented and poorly covered in these databases, limiting their use in cardiac research. A more general limitation is the advanced mathematical and computational skillset required for current data analysis workflows, which presents a barrier for non-specialized groups.

Here, we developed improved data-acquisition workflows coupled to a novel and conceptually-straightforward data analysis strategy to identify alterations in high-MW protein complexes in heterozygous R14^Δ/+^ mouse hearts. Experimental design, methodological improvements and key findings are summarized in Fig 1. Complexome profiling was performed on a cohort of 28wk-old ♂ WT and R14^Δ/+^ mice (Cohort #1, N = 1). At this age, our R14^Δ/+^ mouse model does not exhibit the severe cardiomyopathy, fibrosis and perinuclear aggregate formation observed in end-stage human-patient hearts [7, 41] and thus reflect an early presymptomatic disease state [7, 11] (Fig 1A). Analysis of our CP dataset using conventional complex-centric (via the mCP R-script) and manual candidate-based data analysis approaches (Fig 1C *left and middle*) failed to yield significant insights. We thus developed PERCOM, a novel, conceptually straightforward “protein-centric” analysis workflow that systematic identifies individual proteins with altered elution profiles. Crucially, identification of complexome changes at the level of individual proteins, rather than entire complexes, removes the use of curated protein complexes databases for ground-truth for analysis; thus PERCOM may be highly suited for use in complex tissue types such as myocardium, which, as discussed above, are underrepresented and poorly covered in current protein complex databases (Fig 1C *right*). PERCOM analysis identified changes in supercomplexes involved in mitochondrial membrane organization, oxidative phosphorylation and intercalated disk components important for maintaining intercellular conduction and connectivity (Fig 1D). Alterations in mitochondrial supercomplexes were associated with impaired function and reduced mitochondrial protein abundance. Lastly, altered elution of mitochondrial and ICD complexes was confirmed by performing complexome profiling in a second cohort (Cohort #2) of 9wk-old “juvenile” WT and R14^Δ/+^ mice (N = 1, Fig 1E).

## 2. Results

### 2. 1. Development of a SEC-DIA-MS experimental workflow optimized for cardiac tissue samples

**Fig 2:**
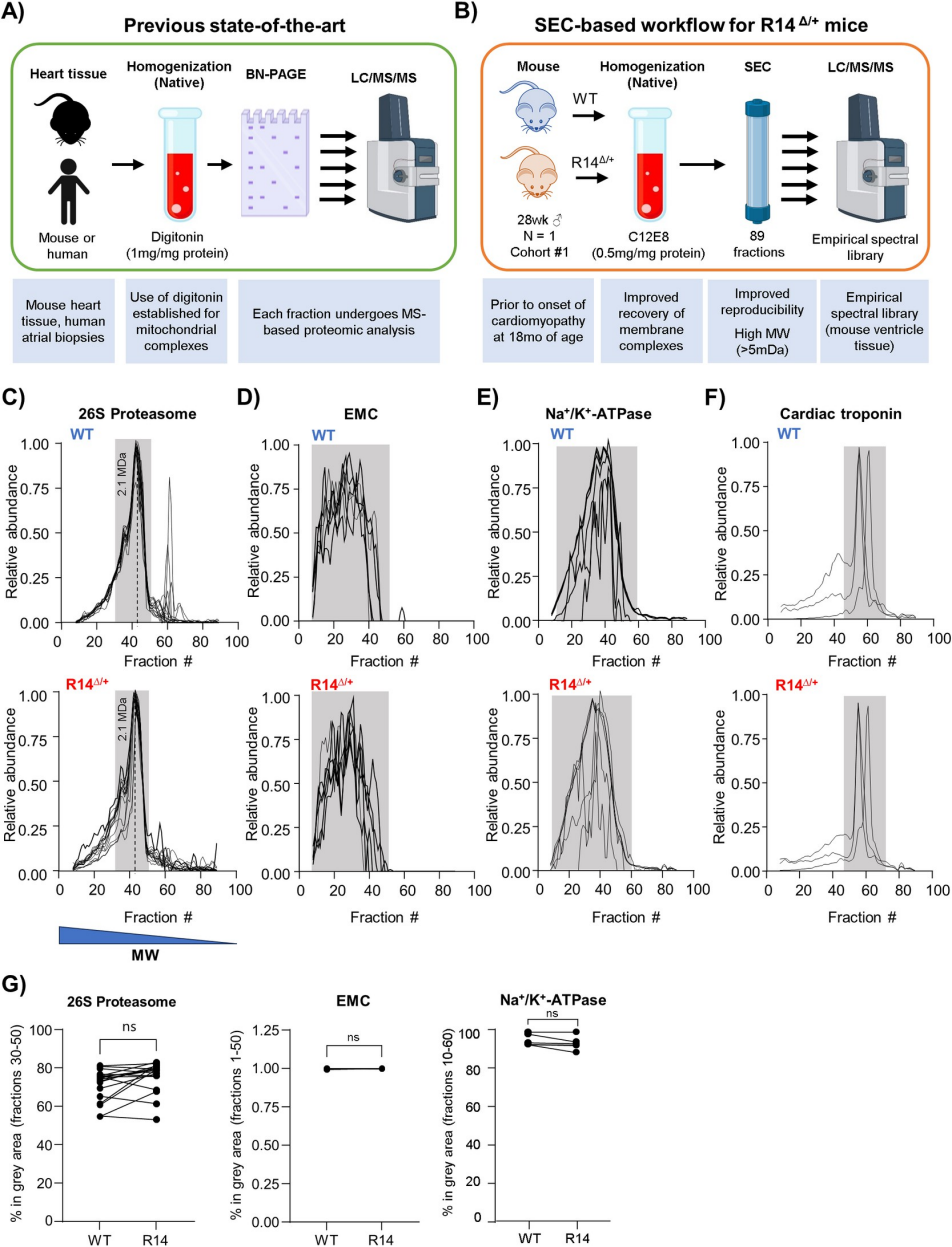
Development of high MW complexome profiling workflows and comparative analysis of WT and presymptomatic R14^Δ/+^-PLN mouse heart tissue. A-B) An improved SEC-MS based complexome profiling workflow for cardiac samples. A) Previous CP studies on mouse and human heart tissue used digitonin and BNE for solubilization and fractionation, respectively. B) Methodological improvements include use of alternative detergents and SEC-based fractionation. For our first experimental cohort (Cohort #1), left-ventricular (LV) tissues were dissected from a single WT mouse and a single sibling-matched presymptomatic 28-week-old ♂ R14^Δ/+^ mouse (N = 1). Membrane fractions were enriched by differential centrifugation, solubilized in C12E8 and separated into 89 fractions by SEC. Homogenization and fractionation workflows were optimized for preservation of membrane protein complexes and detection at high-MW ranges (see text). Each fraction was then subject to MS-based proteomics analysis using a dedicated empirical spectral library for mouse LV tissue for ground-truth. C-F) Sample preparation and SEC-based fractionation workflows preserve protein complexes from diverse subcellular compartments. Constituent proteins of the 26S proteasome (soluble cytosolic complex), ER membrane complex (EMC, integral SER membrane), Na^+^/K^+^-ATPase (integral plasma membrane) and cardiac troponin (sarcomere) were detected from Cohort #1 WT (*top*) and R14^Δ/+^ (*bottom*) mouse LV tissue. Elution profiles represented as a plot of relative abundance vs. fraction #, with each trace representing the profile from a single protein (protein IDs listed in Table 3). Higher fraction # corresponds to lower MW (C, *bottom*). Dotted line: 2.1 MDa MW marker (Full MW calibration in S3 Fig). Peaks corresponding to each complex manually defined (grey underlay). Elution profiles subject to curve smoothing (4th order polynomial, 2nd neighbor). Points lying below the horizontal axis due to smoothing removed for clarity. G) Quantification of complex integrity. The area under the curve of each protein was calculated, and the percent lying within the indicated MW-fraction range plotted. Each paired data point represents the % area-under-curve of a single protein. Significance determined via mixed one-way ANOVA with Šidák corrections for multiple comparison (ns = non-significant).

Conventional CP workflows for cardiac samples typically involve solubilization of mitochondrial or membrane fractions in digitonin, followed by BNE-based fractionation and MS-based protein detection [22, 42–44] (Fig 2A). Key improvements presented in this work include use of a very high MW range (up to ~7.5 MDa) SEC-based fractionation in place of BNE, and selection of an alternate detergent to better preserve membrane protein complexes (Figs 1F and 2B). SEC-based fractionation offers several benefits over BNE, such as enhanced resolution and reproducibility, increased loadability and the option to retain protein complexes in their solubilized native/active state to facilitate additional downstream analysis such as enzyme activity assays or protein/protein crosslinking mass spectrometry (XL-MS) [45]. In particular, enhanced reproducibility is crucial in comparing elution profiles between WT and disease conditions, as in this work, and the increased MW-range facilitates the detection of higher-order assemblies such as the PLN-containing SER Ca^2+^-handling supercomplex with a reported apparent MW of ~5 MDa [22].

We applied this improved workflow to perform SEC-CP on presymptomatic sibling-matched 28wk-old ♂ WT and R14^Δ/+^ mice (Cohort #1, N = 1) as shown in Fig 2B. Snap-frozen left-ventricular (LV)-tissues were gently thawed and carefully homogenized under non-denaturing conditions using a potter homogenizer in detergent-free conditions. Enriched membrane fractions were isolated by differential centrifugation and solubilized in octaethylene glycol monododecyl ether (C12E8, 0.5mg per mg protein) [46]. This homogenization workflow was designed to minimize disruption of potential protein complexes, and has been shown to preserve protein-protein interactions, e.g. the recently discovered interaction between PLN and phosphoadaptor protein 14-3-3 [47] as well as a high-MW SER Ca^+2^-handling supercomplex [22]. The detergent of choice, C12E8, is a non-ionic detergent used in the biophysical characterization of proteins in membrane bilayers [46, 48] and has recently found application in the analysis of protein complexes under near-native conditions by mass spectrometry [49, 50]. The ability to preserve select membrane protein complexes in a functional state for biophysical characterization made this the ideal choice for our study. Indeed, we found that C12E8 was superior to Triton X100, NP40 and Tween-20 for preserving the integrity of membrane protein complexes in cardiac samples (S1 and S2 Figs).

Following solubilization, samples were fractionated into 89 fractions using a novel 1000 Å pore size SEC column chemistry that has recently become commercially available and enables separation of biomolecular assemblies up to 7.5 MDa apparent MW [51] (Bio-SEC-5, 5 μm, 1000 Å; 300x7.8 mm, Agilent, USA). SEC chromatogram and MW-calibration curves for this dataset are shown in S3 Fig. All protein-containing fractions were analyzed by bottom-up data-independent acquisition mass spectrometry (DIA-MS) using a custom annotated MS/MS spectral library to obtain global proteome profiles across the apparent MW dimension. To our knowledge, this is the first successful application a SEC-DIA-MS workflow to myocardial tissue. As a first benchmark, we looked for the 26S proteasome, which is a well-defined ~2 MDa supercomplex. We could resolve a tight peak of 26S proteasomal proteins at the correct MW, validating our sample preparation workflow (Fig 2C). We were also able to resolve many other established protein complexes located in the cytosol (COP9 signalosome), plasma-membrane (Na^+^/K^+^-ATPase, Cav1.2 channel), SER (EMC complex, Sec61) and sarcomere (troponin, titin) as shown in Fig 2D–2F and S3 Fig. Importantly, we did not observe notable changes between WT and R14^Δ/+^ tissue samples, confirming the reproducibility of our workflow (Fig 2G). Complete SEC-MS CP output in S1 Table.

### 2.2. Conventional data analysis strategies fail to yield insights into R14Δ-PLN cardiomyopathy

Conventional strategies for analyzing CP data include manually comparing the elution profiles of known proteins of interest (candidate-based) and systematically quantifying the abundance of known protein complexes that are defined within curated protein complex databases (complex-centric). An overview of these workflows is shown in Fig 1C. We attempted to apply both these workflows to identify changes in the R14^Δ/+^-mice complexome.

#### 2.2.1. The R14Δ-PLN mutation does not alter the elution profile of candidate PLN-interacting proteins

**Fig 3:**
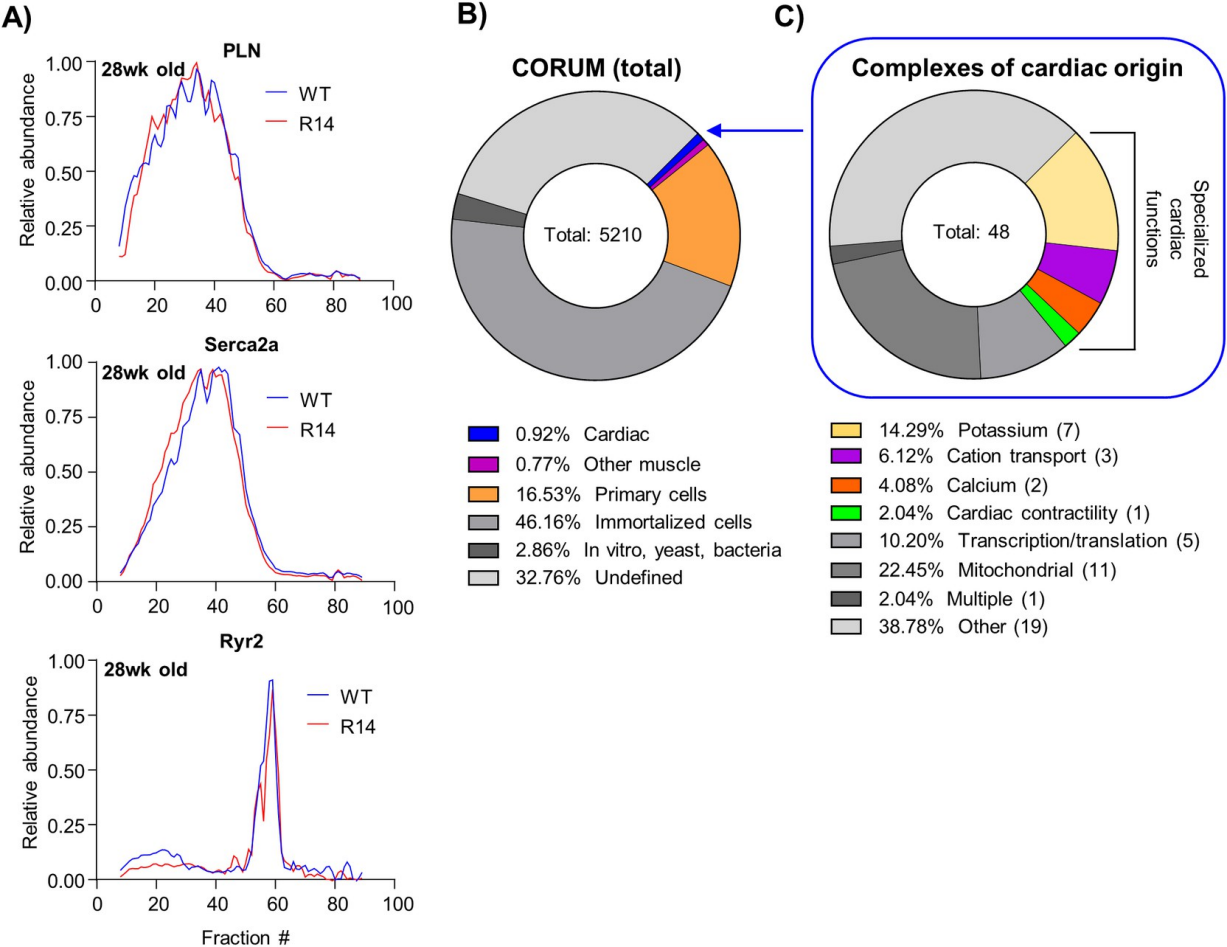
Conventional data analysis workflows fail to yield insights into R14Δ-PLN cardiomyopathy. A) Elution profiles of PLN and associated proteins SERC2a and RyR2 from Cohort #1 CP dataset (28wk old ♂ WT vs. R14^Δ/+^ mouse LV tissue, N = 1, Fig 2B). Elution profiles subject to curve smoothing as in Fig 2B) Origins of all protein complexes documented in the CORUM v. 4.0. database. Database entries were manually annotated based on given source. C) Function of cardiac-origin protein complexes annotated in CORUM. Function assigned based on gene ontology information accompanying each complex entry. Complete annotation of the CORUM database shown in S3 Table.

We previously identified a high-MW (~5 MDa) SER Ca^2+^-handling supercomplex containing PLN, SERCA2a, RyR2, JPH2 (Junctophilin-2, a Cav1.2/RyR2 and SER tethering protein) and PPP1R3A (a protein phosphatase 1 targeting protein) present in both mouse-heart and human-atrial tissues [22]. This PLN-containing supercomplex, with a clear mechanistic link to the R14Δ-PLN Ca^2+^-cycling defect, is the obvious candidate for interrogation. We were able to robustly detect the SER Ca^2+^-handling supercomplex in our Cohort #1 CP datasets (WT vs. R14^Δ/+^, 28wk-old ♂, N = 1, Fig 2B); however, no notable difference was found between the elution profiles of PLN, SERCA2a and RyR2 from WT and R14^Δ/+^ mice (Fig 3A). Orthogonal evaluation was performed via STED super-resolution microscopy of PLN-SERCA2a and PLN-RyR2 colocalization. Consistent with our previous findings [22], significant PLN/SERCA2a and PLN/RyR2 overlap was observed; however, colocalization was not significantly altered in R14^Δ/+^ mice (S4 Fig). Recent work suggests that the R14Δ mutation alters the interaction between PLN and known binding partners such as HAX-1, HRC and SERCA2a [9]. In addition, an increased stability of the PLN-R14Δ pentamer has also been reported in HEK293 cells overexpressing PLN [52]. While these prior findings indicate changes at the level of individual binding interactions, these do not appear to translate into observable disruption of the functional supercomplex.

#### 2.2.2. Current systematic analysis workflows are poorly suited to specialized cell types such as cardiomyocytes

The complex cellular phenotype associated with R14Δ-PLN, which includes impaired ER-mitochondrial contact [53], mitochondrial dysregulation [14], formation of PLN-positive perinuclear aggregates [7, 12] and UPR signaling [13] suggests alterations in other protein complexes/supercomplexes that may be functionally and/or spatially remote from the PLN-containing SER-Ca^2+^-handling supercomplex. This, in conjunction with the lack of changes in functionally/molecularly adjacent candidates describe above suggest a need for systematic analysis of our CP dataset. Such analysis generally involves the use of software packages (e.g. mCP [33], CCprofiler [34] or ComplexBrowser [36]) that evaluate the presence and abundance of known annotated protein complexes based on the coelution of known subunits, with ground truth provided by external annotated protein databases such as ComplexPortal [38] or CORUM [37]. While this approach has been used with success to identify changes in well-established protein complexes, such as the respiratory chain complexes I-V in disease states [54], several shortcomings limit their application to this study:

1. Protein complex databases document well-defined and established complexes such as the T-cell receptor complex [55], K^+^-channel tetramers [20] and the Cop9 signalosome [56]; however, higher-order supercomplexes, which may have poorly-defined and fluid stoichiometry and/or composition (e.g. the aforementioned SER Ca^2+^-handling supercomplex [22], intercalated disks components [57] and signaling microdomains [58]), may not be represented.
2. These databases largely represent data from non-cardiac cell lines. For example: less than 1% of CORUM 4.0 entries are derived from primary cardiac material (Fig 3B), and only ~25% of those (representing 13 entries in total) are associated with cardiac-specific functions such as ion channel activity, as opposed to general cellular functions such as mitochondrial function, transcriptional regulation, etc. (Fig 3C and S3 Table). As a consequence, many key cardiac-specific proteins (such as JPH2) and supercomplexes (such as the SER Ca^2+^-handling supercomplex [22]) are not covered in either the ComplexPortal or CORUM databases.
3. More generally, these databases represent only a subset of the human proteome (22% for CORUM 4.0 [37]).
4. Lastly, these packages frequently utilize advanced mathematical and statistical methods, often executed as custom scripts or programs. Thus, these strategies generally require specialized computer programming and bioinformatics expertise for optimal implementation, which presents an accessibility barrier more widespread adoption.

Taken together, these targeted complex-centric analysis strategies may be restrictive to a narrow subset of well-established protein complexes curated from non-cardiac cells, and thus fail to detect changes in cardiac-specific complexes and/or less well-defined higher-order supercomplexes. Furthermore, we also perceive a pressing need for a conceptually straightforward approach, which can be easily accessed by non-specialized working groups within the greater research community.

To illustrate these limitations, we attempted to apply traditional database-dependent data analysis workflows to our 28wk-old mouse Cohort #1 dataset (WT vs. R14^Δ/+^, ♂, N = 1, Fig 2B). We employed the mCP R-software package [33] to identify and quantify, within our CP dataset, the abundance of protein complexes defined by the CORUM 4.0 protein complex database [37]. This workflow was able to identify and quantify the abundance of 57 annotated protein complexes in both WT and R14^Δ/+^ cardiac samples (S5 Fig). Nonetheless, the majority of detected complexes were not cardiac specific and, vice-versa, several noteworthy cardiac-specific complexes such the SER Ca^2+^-handling supercomplex were not identified, despite the constituent components (e.g. PLN and SERCA2a) co-eluting within our dataset (Fig 3A). Lastly, higher-order supercomplexes, such as the well-characterized respiratory supercomplex (RSC) containing mitochondrial complexes CI, III and IV [59], were not detected, despite strong co-elution and detection of the individual constituent complexes (discussed later in this work).

### 2.3. PERCOM: An accessible, conceptually straightforward strategy for systematic analysis of CP data

To overcome these limitations, we developed PERCOM, a novel “protein-centric” analysis workflow with two main objectives: 1) to enable identification of individual proteins with altered elution profiles (as opposed to established and annotated protein complexes) in order to avoid the limitations of using protein complex databases, and 2) to be conceptually straightforward, accessible to the general research community, and, ideally, be implemented using common commands and functions available on ubiquitous data processing software (e.g. Microsoft Excel, Fig 1C).

#### 2.3.1. Conceptual basis of PERCOM

**Fig 4:**
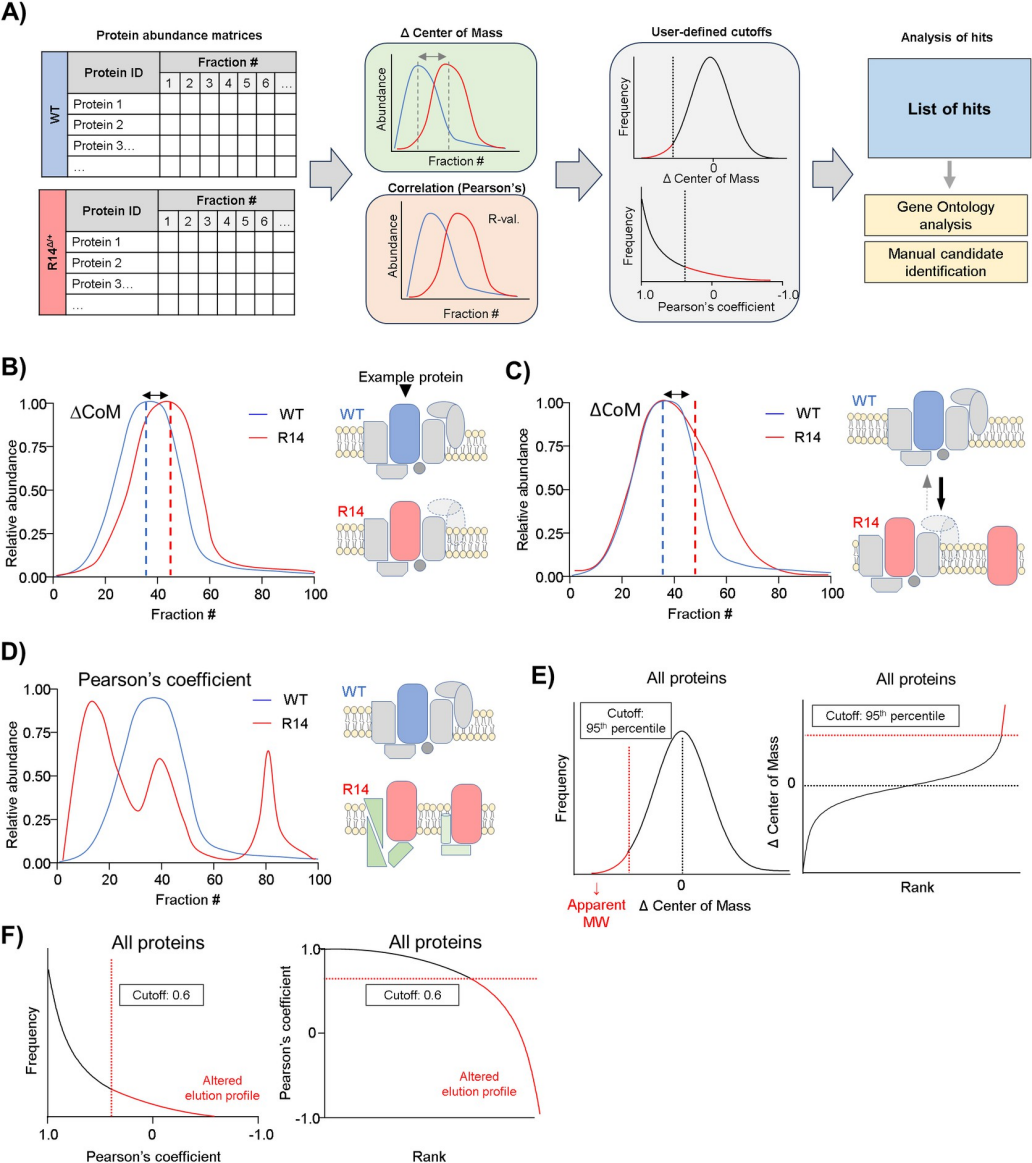
PERCOM, an unbiased and easily-accessible protein-centric analysis workflow. A) Graphical illustration of the PERCOM data analysis strategy using hypothetical example data. Input formatted as a matrix of protein abundances where protein ID and fraction number are organized by row and column, respectively, and each cell contains a value representing protein abundance. Each condition is represented by an individual matrix: in this case, WT and R14^Δ/+^. Two parameters are analyzed: the change (Δ) of the center of mass (CoM) and Pearson’s correlation coefficient (R-value). Quantitative cutoffs are then applied to identify hits, which may then be subject to candidate-based analysis of individual proteins of interest or gene-ontology analysis to identify overrepresented cellular components and/or biological pathways. B-C) Potential changes in supercomplex composition/integrity detected by ΔCoM. Here, a hypothetical protein forms a supercomplex with CoM at fraction 40 in WT mice (blue). A change in CoM due to a horizontal shift of the elution profile in R14^Δ/+^ mice (red) may indicate the loss of one or more subunits, affecting the apparent MW of the entire supercomplex population (B). In contrast, a change in CoM due to skewing of the elution profile towards lower-MW fraction (C) may indicate an altered equilibrium between a fully-assembled supercomplex and intermediate assemblies or monomers in a subpopulation. D) Potential changes in supercomplex composition/integrity detected by Pearson’s correlation. A low correlation coefficient may indicate gross changes in supercomplex composition. E-F) Expected probability distributions and cutoffs used in this study. ΔCoM is expected to be normally distributed with a mean of 0 (no change, black dotted line). Probability distribution thus might be approximated with a Gaussian function, and rank-plot approximated with a cumulative Gaussian function (E). A 95^th^ percentile cutoff was chosen for this study (red dotted line). As this study is interested in disruption of high-MW protein complexes, which presumably manifest as a decrease in overall MW as illustrated in B-C), hits with increased CoM are not considered. Probability distribution of Pearson’s correlation expected to follow an exponential decay function, and rank-plot expected to follow a log-decay function (F). A Pearson’s coefficient cutoff of ≥0.6 was chosen for this study (red dotted line). All data shown in this figure is hypothetical.

The PERCOM data analysis workflow is summarized in Fig 4A. Input consist of one matrix of protein abundances (either raw or relative intensities) per condition (in this case: the WT and R14^Δ/+^ mouse heart from Cohort #1). Protein ID and fraction number are organized by row and column, respectively. The elution profiles of each protein are then compared based on two parameters: Pearson’s coefficient of correlation (R-value) and the center-of-mass (CoM), giving rise to the name: PERCOM. Both CoM and Pearson’s score can be visualized on a protein elution plot of protein abundance vs. fraction number (Fig 4B–4D). CoM is defined as the fraction which evenly divides the area-under-the-curve (50% on either side), and the change in CoM (ΔCoM) represents the difference in CoM between two conditions (in this case, WT and R14^Δ/+^). The combination of ΔCoM and Pearson’s coefficient was chosen to maximize detection of proteins with altered distribution profiles in a complementary manner. We postulate that ΔCoM would detect proteins with elution peaks shifted or skewed towards lower-MW fractions, which may indicate subtle changes in supercomplex composition and/or assembly efficiency (Fig 4B and 4C). In contrast, Pearson’s coefficient would detect proteins with significant changes in elution profile shape, which may be consistent with complete disruption or reorganization of a supercomplex (Fig 4D). We work under the assumption that ΔCoM will be approximately normally distributed (Fig 4E) with a mean value of 0 (i.e. no change), whereas Pearson’s coefficient is expected to follow an approximately exponential-decay probability distribution (Fig 4F). Hits are defined based on quantitiative cutoffs for both ΔCoM and Pearson’s coefficient. These hits can then be manually curated to identify potential proteins of interest (e.g. based on known functional or physical interaction or disease relevance), or gene-ontology analysis to identify statistically overrepresented cellular components or molecular pathways (Fig 1C and Fig 4A).

#### 2.3.2. Application of PERCOM to 28wk-old WT and R14^Δ/+^ mouse ventricular tissue

**Fig 5:**
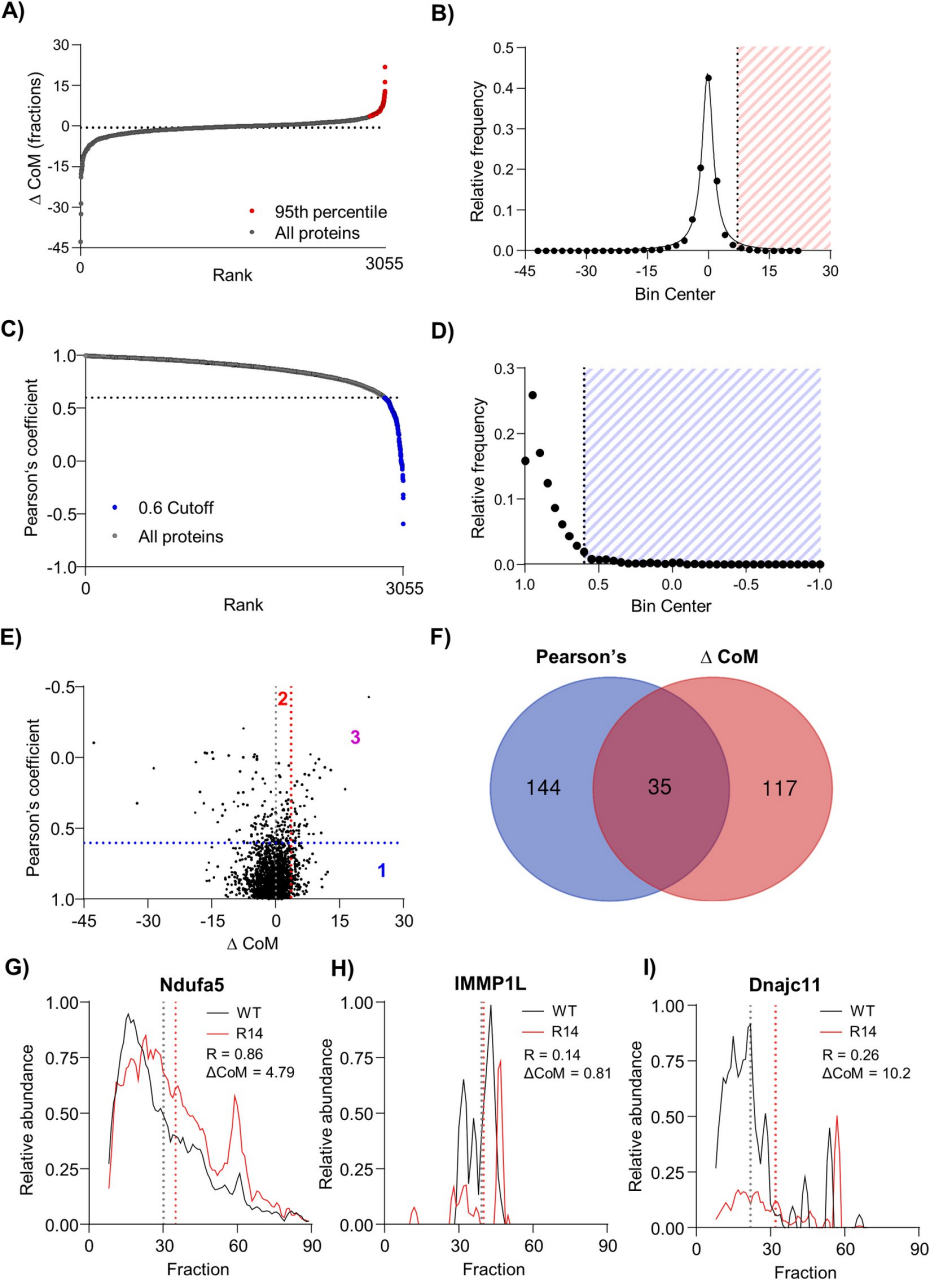
PERCOM identifies proteins with altered MW-profiles in adult 28wk-old R14^Δ/+^ mice. A-B) ΔCoM analysis of Cohort #1 CP dataset (28wk old ♂ WT vs. R14^Δ/+^ mouse LV tissue, N = 1, Fig 2B). Results represented as rank-plot (*left*) and distribution histogram (*right*). Distribution can be approximated by a Gaussian-like (Cauchy) distribution function (solid line). 95^th^ percentile threshold indicated in red yielded 177 hits out of 3055 proteins detected in both WT and R14^Δ/+^ tissue. C-D) Analysis based on Pearson’s coefficient of correlation. Results represented as rank-plot (*left*) and distribution histogram (*right*). Hits (Pearson’s correlation <0.6) indicated in blue: 144 proteins out of 3055 detected. E) Orthogonal plot of Pearson correlation vs. ΔCoM. Cutoffs indicated with dotted line. 3 quadrants are identified: hits with altered CoM (1), hits with Pearson > 0.6 (2), and hits satisfying both criteria (3). F) Venn diagram of hits. G) Example elution profiles for a protein with altered CoM (Ndufa5), a protein with Pearson’s coefficient <0.6 (IMMP2L), and a protein meeting both criteria (Dnajc11). CoM indicated by dotted lines, and Pearson’s correlation coefficient (R) and ΔCoM shown. Elution profiles subject to curve smoothing as in Fig 2.

We applied PERCOM to identify proteins with altered distribution in our Cohort #1 CP dataset (WT vs. R14^Δ/+^, 28wk-old ♂, N = 1, Fig 2B). As expected, the frequency distribution of ΔCoM was centered near 0 and followed a Gaussian-like (Cauchy) distribution (Fig 5A and 5B) while Pearson’s coefficient followed an exponential decay frequency distribution (Fig 5C and 5D). A 95^th^ percentile cutoff (corresponding to ~2x standard deviations from the mean assuming approximate Gaussian distribution) was used for ΔCoM, and a cutoff of ≥0.6 was used for Pearson’s coefficient: similar cutoffs are used in established workflows to detect complexes *de novo* by coelution [39]. We identified 296 proteins hits (out of a total of 3055 detected in both WT and R14^Δ/+^ tissue): 117 with ΔCoM, 144 with Pearson’s, and 35 with both (Fig 5E and 5F). Full list of PERCOM hits from Cohort #1 CP dataset available in S4 Table. As expected, ΔCoM proved generally suitable for identifying proteins with elution profiles shifted towards lower-MW fractions, and Pearson’s correlation for proteins with altered elution profile shapes (Fig 5G–5I).

Assessing technical and biological reproducibility between control and experimental samples in workflows involving a high degree of fractionation such as this one can be complicated by the fact that a subset of proteins/genes are expected to be altered between the experimental and control conditions, with the clear requirement to exclude these from any analysis of reproducibility. In addition to providing a conceptually-straightforward workflow to quantitatively identify proteins of interest, the reciprocal information (proteins with unaltered elution profiles) represents an unbiased and quantitative approach to identifying a panel of “unchanged” proteins to verify technical reproducibility between samples. The elution profiles of “non-hits” were sorted by hierarchical clustering and represented as heat-maps (S6 Fig). This heatmap analysis is an established method for evaluating reproducibility of complexome-profiling experiments [60, 61], and supports high reproducibility of our workflow.

### 2.4. PERCOM identifies alterations in mitochondrial supercomplexes in presymptomatic 28wk-old R14^Δ/+^ mice

#### 2.4.1. PERCOM identifies several inner-mitochondrial membrane proteins with altered elution profiles in adult 28wk-old R14^Δ/+^ mice

Gene ontology analysis of PERCOM hits from our Cohort #1 CP dataset showed a statistically-significant enrichment of mitochondrial components (Table 1). Among affected components is the mitochondrial respirasome supercomplex (RSC) containing mitochondrial complexes CI, CIII and CIV. The coupling of mitochondrial CI, CIII and CIV proton pumping activity in RSCs allows for increased metabolic efficiency with reduced ROS production [62, 63]. In cardiomyocytes, RSC assembly plays an important role in sustaining a high level of ATP synthesis and maintaining energy balance [64]. Conversely, loss of RSC integrity is associated with cardiomyopathies including DCM [65], heart failure [66] and ischemia-reperfusion injury [67].

**Table 1:** Gene ontology analysis of PERCOM hits from Cohort #1 28wk-old WT vs. R14^Δ/+^ mice CP dataset.

**1) Change in Center of Mass (CoM)**			
**GO cellular component**	**Fold enriched**	**FDR**	**Examples**
MICOS complex	92.17	2.14E-05	MICOS complex subunit Mic27, Mic19, Mic10, Dnajc11
SAM complex	82.95	1.87E-02	Samm50, Dnajc11
HFE-transferrin receptor complex	69.13	2.43E-02	Serotransferrin, Transferrin receptor protein 1
ribosomal subunit	17.99	2.57E-13	40S ribosomal proteins S2, S3, S8, etc. 60S ribosomal proteins L24, L14, L11, etc.
Mitochondrial Respirasome	12.96	5.11E-04	Uqcr11, Ndufs6, Ndufa5, Uqcc3
caveola	12.70	5.57E-04	Flotillin-1 and 2, Caveolin-1 and 2, Cavin-4, Nitric oxide synthase (endothelial)
**2) Pearson’s correlation coefficient**			
**GO cellular component**	**Fold enriched**	**FDR**	**Examples**
mitochondrial inner membrane peptidase complex	> 100	2.00E-02	Mitochondrial inner membrane protease subunit 1 and 2
Desmosome	29.07	2.26E-03	Desmin, Desmoplakin, Desmoglein-1-alpha, Plakophilin-1
Nucleosome	19.96	2.07E-07	Histone proteins (H2bc9, H4f16, H2ax, etc.)
cytosolic large ribosomal subunit	19.57	3.36E-05	60S ribosomal proteins (L14, L27a, L11, etc.)

Gene ontology analysis of PERCOM hits from Cohort #1 CP dataset (28wk old ♂ WT vs. R14^Δ/+^ mouse LV tissue, N = 1, Fig 2B). Hits with altered CoM and Pearson’s coefficient subject to separate analysis and shown in parts 1) and 2) of the table, respectively. Mitochondrial and intercalated disk components highlighted in orange and green, respectively. Gene Ontology based on biological component performed by Panther version 18 [68]_._ Statistical significance determined based on corrected false discovery rate (FDR) below 5% (0.05).

#### 2.4.2. The mitochondrial CI/III/IV respirasome effecting efficient proton pumping is altered in 28wk-old R14^Δ/+^ mice

**Fig 6:**
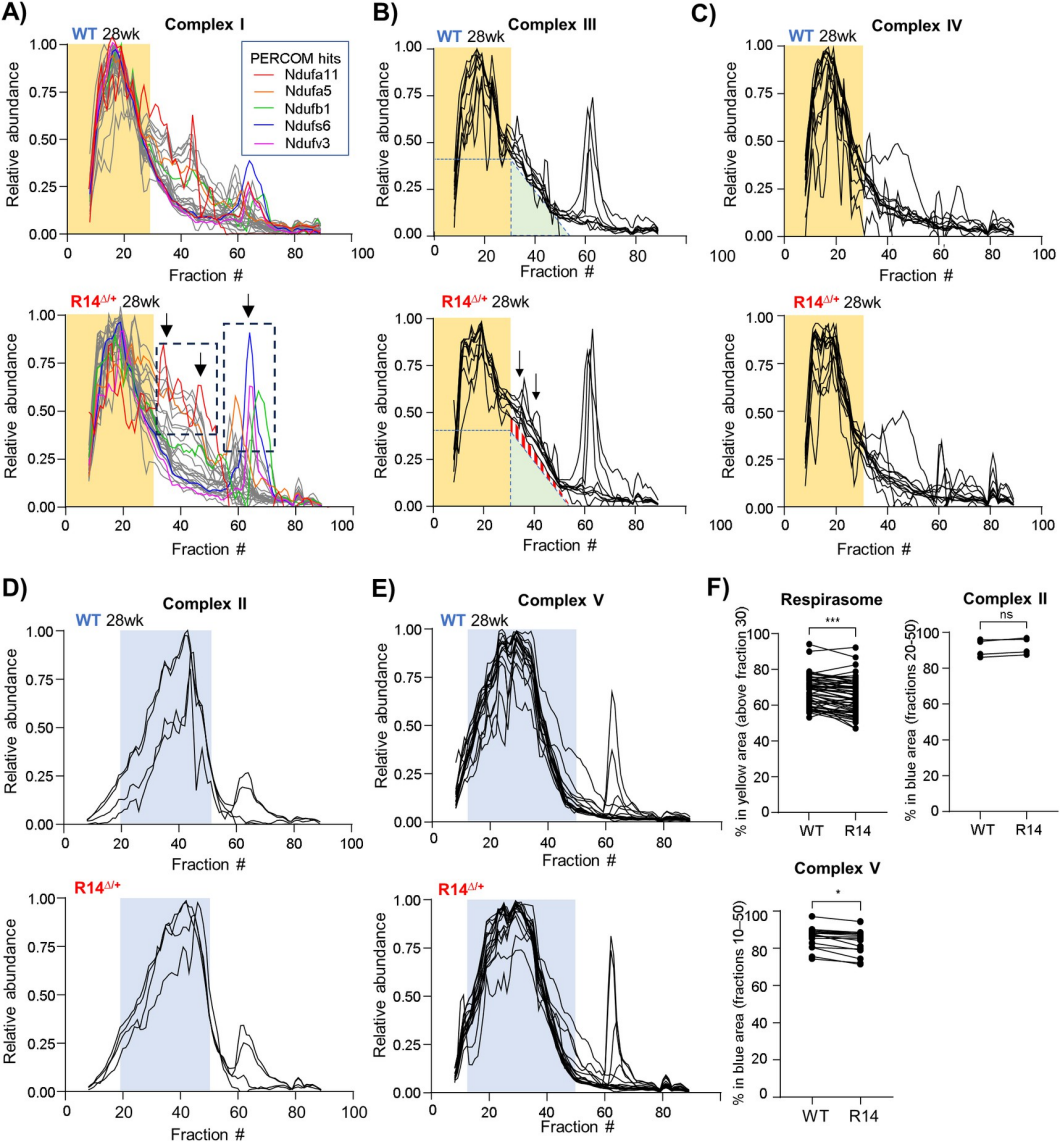
Alterations to mitochondrial respirasome supercomplexes in adult 28wk-old R14^Δ/+^ mice. A-C) Elution profiles of respirasome components from Cohort #1 CP dataset (28wk old ♂ WT vs. R14^Δ/+^ mouse LV tissue, N = 1, Fig 2B). Each trace represents the elution profile of a single protein (protein IDs listed in Table 3). The respiratory supercomplex (RSC) contains mitochondrial complexes CI, CIII and CIV, and occupies a high-MW peak between fractions #0 and #30 (indicated with yellow underlay). A) Elution profiles of mitochondrial complex I proteins. Proteins identified as PERCOM hits shown in color. Areas of notable difference between WT (*top*) and R14^Δ/+^ mice (*below*) indicated with arrows and dotted boxes. B) Elution profiles of mitochondrial complex III proteins. In addition to the RSC peak (yellow underlay), CIII proteins also form an intermediate-MW “tail” in WT mice (green triangle). This “tail” is more pronounced in R14^Δ/+^ mice (red triangle, arrows). C) Elution profiles of Complex IV proteins presented as in A). D-E) Elution profiles of mitochondrial complexes CII and CIV not part of the RSC. Both complexes form lower-MW peaks (blue underlay) that is distinct in both apparent MW and shape from the RSC-peak shown in A-C). F) Quantification of mitochondrial respiratory complex integrity. The area under the curve of each protein comprising the respirasome (complex CI/III/IV) and non-respirasome complexes CII and CV was calculated, and the percent lying within the indicated MW-fraction range plotted. Each paired data point represents the % area-under-curve of a single protein. MW-fraction ranges correspond to the yellow and blue color underlays in panels A-E. Respirasome components (CI/III/IV) and CV showed significant disruption. In contrast, CII (F), as well as unrelated protein complexes such as the 26s proteasome, EMC and Na^+^/K^+^-ATPase remained unaffected (Fig 2). Significance determined via mixed one-way ANOVA with Šidák corrections for multiple comparison (***p < 0.001, *p < 0.05, ns = non-significant). Multiple comparison testing encompassed 26S proteasome, EMC and Na^+^/K^+^-ATPase profiles shown in Fig 2. All elution profiles subject to curve smoothing as in Fig 2.

To visualize these potential RSC changes, we compared the elution profiles of the individual proteins comprising mitochondrial complexes CI-V in our Cohort #1 CP dataset. Constituent proteins of mitochondrial complexes CI, III and IV, but not II and V, co-eluted in a broad ≥2 MDa high-MW peak (Fig 6A–6E, fractions 1–30, yellow underlay), which we interpret to correspond to the RSC. Several CI proteins were identified as PERCOM hits (S4 Table) and, we found the general elution profile for many CI proteins to be visibly disrupted in R14^Δ/+^ mice (Fig 6A, dashed boxes and arrows). The profile of CIII proteins was also shifted slightly towards lower MW-fractions (Fig 6B, red hashmarks and arrows). Complex CIV remained visually unchanged (Fig 6C). To quantify these changes, we calculated the percentage of each protein within a certain MW fraction range. Ranges were chosen based on the apparent elution profile peaks in the WT-mouse sample: between fractions #0 and #30 for RSC components (CI, CIII and CIV), and between fractions #20 and #50 and fractions #10 and #50 for CII and CIV, respectively. We found a significant decrease in the percentage of RSC component proteins migrating within fractions #0 to #30 in the R14^Δ/+^ mouse (Fig 6F). A similar effect was noted for CV, but not CII (Fig 6F). In contrast, the elution profiles for unrelated proteins such as the plasma-membrane Na^+^/K^+^-ATPase, EMC and 26S proteasome were unchanged (Fig 2C–2G).

#### 2.4.3. A putative mitochondrial MICOS/TOM/SAM supercomplex involved in inner-mitochondrial membrane organization is altered in 28wk-old R14^Δ/+^ mice

In addition to the RSC, PERCOM analysis of our Cohort #1 CP dataset identified several other components involved in mitochondrial organization and protein import/sorting (Table 1). Of particular interest is the mitochondrial contact site and cristae organizing system (MICOS) complex. MICOS is an evolutionarily conserved structure that facilitates mitochondrial organization. It is located primarily at cristae junctions (CJs) of the inner mitochondrial membrane (IMM) [69] where it plays a critical role in CJ formation by providing membrane curvature [70]. It also acts as a hub for protein import and sorting through physical interactions with several other protein complexes, including the translocase of the outer mitochondrial membrane (TOM) complex (responsible for protein import through the OMM) [71, 72] and the sorting and assembly machinery (SAM) complex (responsible for membrane insertion of OMM proteins) [73]. Lastly, it is involved in the organization of respiratory complexes into the RSC [74, 75]. This last point is of particular interest, as disruptions in the MICOS complex may be functionally associated with the changes in the RSC discussed in the previous section.

**Fig 7:**
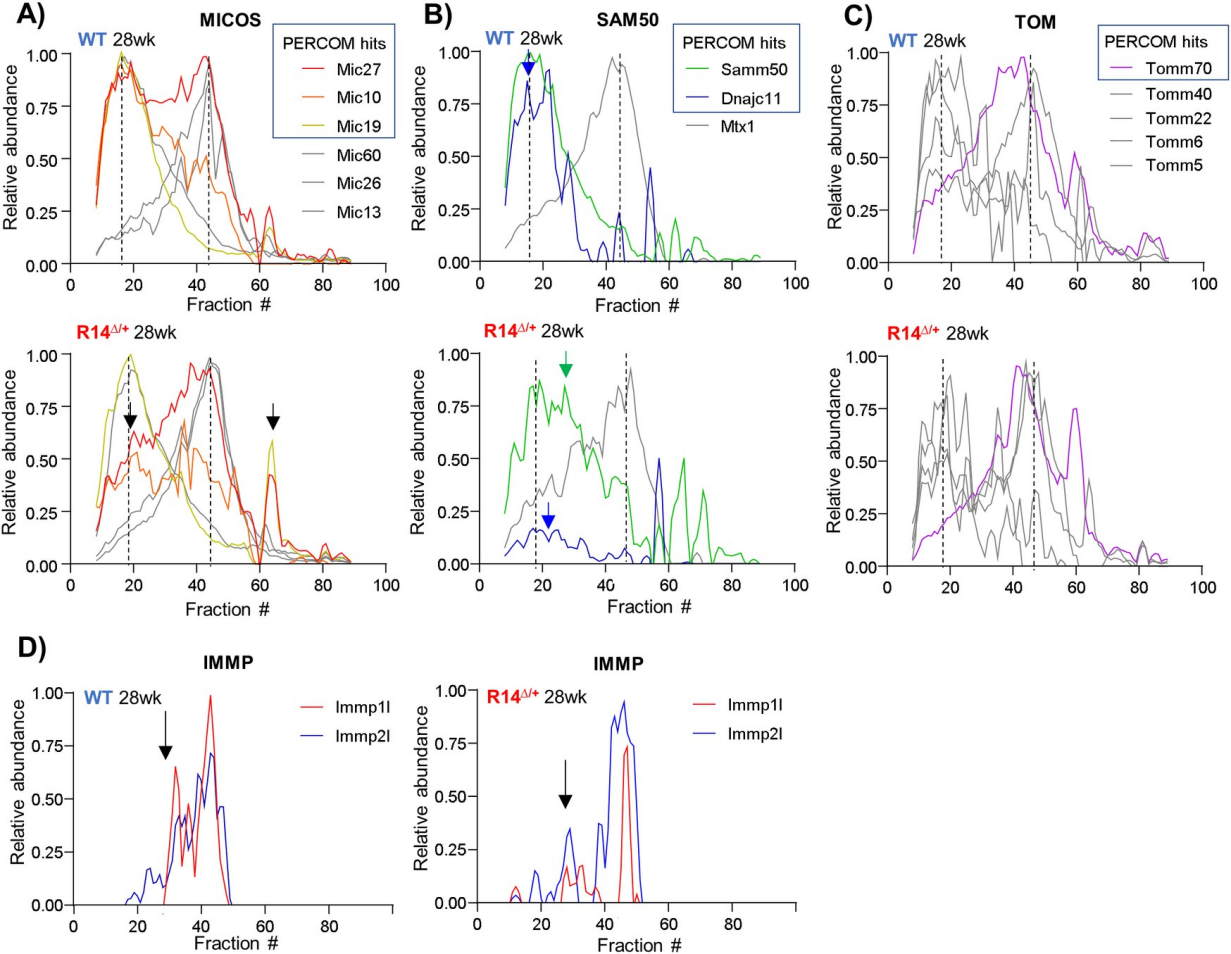
Alterations to protein complexes involved in mitochondrial organization in28wk-old R14^Δ/+^- mice. A-C) A putative MICOS/SAM/TOM supercomplex is disrupted in adult 28wk-old R14^Δ/+^ mice. Elution profiles of proteins comprising the MICOS, SAM50 and TOM complexes from Cohort #1 CP dataset (28wk old ♂ WT vs. R14^Δ/+^ mouse LV tissue, N = 1, Fig 2B). All components at-least partially co-elute as two high-MW peaks (dotted vertical lines), suggesting that MICOS, SAM50 and TOM associate as a supercomplex. PERCOM hits indicated with colored lines; unaltered proteins in gray. Areas of notable difference indicated by arrows. D) Alterations to the inner mitochondrial membrane peptidase (IMMP) complex in adult 28 wk-old R14^Δ/+^ mice. The IMMP complex consists of two proteins (Immp1l and Immp2l), both of which were identified as PERCOM hits (red and blue traces, respectively). Areas of notable difference indicated by arrows. Immp1l trace shown previously in Fig 5. All elution profiles subject to curve smoothing as in Fig 2.

To-date, two MICOS subcomplexes have been identified in yeast: a “core component” containing Mic60 oligomers and accessory subunit Mic19 [72], and a “Mic27 subcomplex” containing Mic10, Mic12, Mic26 and Mic27 [76]. To visualize potential changes in MICOS complex assembly and/or composition, we compared the elution profiles of these components in our Cohort #1 CP dataset (Fig 7A). We found that the Mic27 subcomplex was visibly altered, with 3 out of 4 detected components identified as PERCOM hits (Mic10, Mic12, Mic27, but not Mic26). In addition, Mic19, but not the core Mic60 protein was also affected. Interestingly, assembly of the Mic27 subcomplex is dependent on ER-mitochondrial contact sites, whereas self-assembly of Mic60 oligomers occurs independently of both Mic19 and ER–mitochondria contact [77]. Previous work has shown that the R14Δ-PLN negatively impacts ER-mitochondrial contact sites in cardiomyocytes differentiated from R14^Δ/+^ hiPSC [53]. Whether ER-mitochondrial contact sites are disrupted in adult transgenic mice remains unknown; however, this would be consistent with our finding of altered Mic27 subcomplex assembly, but not Mic60 oligomerization.

The MICOS complex co-ordinates with other import/organizational machinery such as the TOM [71, 72] and SAM [73] complexes. MICOS, SAM and TOM components at-least partially co-eluted in both WT and R14^Δ/+^ mice (Fig 7A–7C), consistent with the formation of a higher-order MICOS/SAM/TOM supercomplex [78]. Notably, components of the SAM complex were identified as being statistically enriched among our PERCOM hits (Table 1), raising the possibility that R14Δ-PLN may alter not only the MICOS complex, but also this putative MICOS/SAM/TOM supercomplex. Indeed, the elution profiles of both SAM and TOM components were altered in our Cohort #1 CP dataset (Fig 7B and 7C). Lastly, we examined the inner mitochondrial membrane peptidase (IMMP) complex. This assembly is responsible for proteolytic maturation of proteins targeted to the intermembrane space [79]. The IMMP complex was identified as a PERCOM hit (Table 1), and both IMMP components (IMMP1 and IMMP2) displayed radically altered elution profiles in 28wk-old R14^Δ/+^ mice (Fig 7D). We speculate that changes to these complexes involved in mitochondrial organization and protein import may contribute to the overall mitochondrial alterations observed in R14^Δ/+^ mice.

### 2.5. Mitochondrial supercomplex alterations are associated with reduced mitochondrial function and proteomics changes

**Fig 8:**
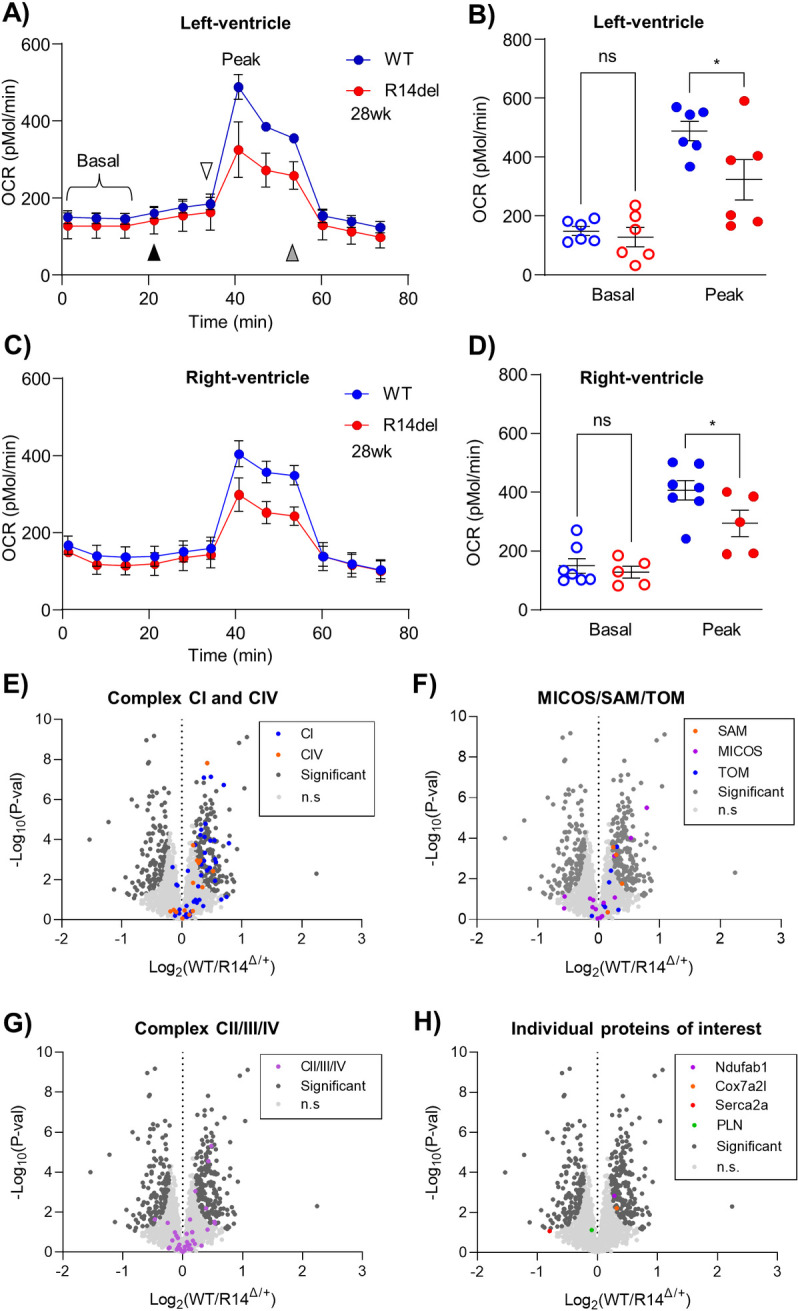
Reduced mitochondrial function and protein expression in 21-28wk-old R14^Δ/+^ mice. A-D) Mitochondrial function measured in left- and right-ventricular cardiomyocytes isolated from 28wk-old ♂ WT and R14^Δ/+^ mice (N = 5–7). Note that these mice are from a separate independent cohort from those used for complexome profiling. Oxygen consumption rate (OCR) measured by Seahorse under basal conditions in media containing pyruvate (1 mM) and glucose (10 mM), and peak OCR measured following addition of CCCP (1.5 μM, hollow arrow). Oligomycin (3 μM, black arrow) and Antimycin/Rotenone (0.5 μM each, gray arrow) were added in an attempt to assess OCR due to proton leak and non-mitochondrial sources, respectively. Note that it is not unusual to observe little/no change in the OCR of cardiac cells following addition of Oligomycin (see text). P-values determined by one-way ANOVA with Šidák correction for multiple comparison. * p < 0.05, ns = non-significant. E-H) Global protein expression assessed in 21 wk-old ♂ WT and R14^Δ/+^ mice via bottom-up DIA-MS proteomics workflows (N = 4). Again, note that these mice are from a separate independent cohort from those used for complexome profiling. Protein expression represented as a Volcano plot. Proteins with significant and non-significant (ns) changes in expression indicated with dark- and light-grey dots, respectively. E) Reduced expression of mitochondrial complex CI and CIV components (blue and orange, respectively). CI and CIV components were found to be significantly overrepresented amongst depleted proteins (Table 2). F) Decreased expression of proteins involved in inner-mitochondrial membrane (IMM) organization. G) Expression of targeted candidate proteins of interest: Ndufab1 (purple), Cox7a2l (orange), SERCA2a (red) and PLN (green). H) As a negative control, expression of mitochondrial complex CII, CIII and CV components (purple) remains unchanged, supporting specificity of our observed changes. Cellular component annotation retrieved from UniprotKB.

We next explored whether these changes to mitochondrial supercomplexes are associated with impaired mitochondrial function. Mitochondrial function was evaluated using the Seahorse assay. Oxygen consumption rates (OCRs) were measured in media containing pyruvate (1 mM) and glucose (10 mM) at both basal levels and following addition of Oligomycin (3 μM), CCCP (1.5 μM), and Antimycin/Rotenone (0.5 μM each). While basal oxygen consumption rates (OCRs) were not significantly decreased, maximal OCR was significantly reduced in left- and right-ventricular cardiomyocytes isolated from 28wk-old R14^Δ/+^ mice (Fig 8A–8D), consistent with recent reports in 8-12wk-old mice [14]. Note that previous reports have also reported little/no change in cardiac cells following addition of Oligomycin [80].

In addition, we sought to correlate the observed changes in mitochondrial supercomplex composition (Figs 6 and 7) and mitochondrial function (Fig 8A–8D) with changes at the proteome level. LV-tissues from 21wk-week-old WT and R14^Δ/+^ mice (N = 4) were subjected to MS-based proteomics analysis using a tandem mass tag (TMT)-based isotopic labeling workflow for normalization. 3422 proteins were detected in this experiment, with 194 being significantly depleted in R14^Δ/+^ mice (Fig 8E–8H). Gene ontology analysis on these depleted proteins revealed a statistically significant overrepresentation of RSC components (respiratory complex I and IV, Fig 8E, Table 2); similar statistical analysis on proteins enriched in R14^Δ/+^ mice LV tissue yielded no results (Table 2). The reduction in RSC component abundances mirrors our observation of altered RSC elution profiles (Fig 6). Furthermore, we also observed a significant depletion in MICOS, SAM, and TOM complex protein expression (Fig 8F), which is consistent with their altered elution profiles as observed by complexome profiling (Fig 7). In contrast, mitochondrial complexes CII, CIII and CV were not altered (Fig 8G), supporting specificity of mitochondrial RSC complex alterations. Lastly, we performed a targeted analysis of proteomics hits (significantly up- or downregulated proteins) to identify specific candidates of interest. NDUFAB1 and COX7A2L play key roles in RSC assembly [65, 81–84]. Both of these proteins localize to the inner mitochondrial membrane and act as assembly factors of mitochondrial complex CI and CIII, respectively; loss of expression of either protein has been shown to disrupt mitochondrial respirasome assembly/integrity, reduce mitochondria function and increase ROS production in cardiac systems [65, 85]. Interestingly, expression of both of these assembly factors was significantly reduced in R14^Δ/+^ mice (Fig 8H). Given the established role of both these proteins in respiratory supercomplex assembly and mitochondrial function, it is interesting to speculate if/how their reduced expression in R14^Δ/+^ mice may be functionally linked to altered mitochondrial supercomplex assembly and, ultimately, metabolic function. As an interesting footnote, we observed an increase in expression of the SERCA2a SER Ca^+2^-reuptake pump in R14^Δ/+^ mice (Fig 8H): another candidate protein of interest that is both functionally and physically associated with PLN. Increased SERCA2a expression is consistent with studies showing a hyperdynamic intracellular Ca^2+^ signaling and increased diastolic Ca^2+^ [86], but is in contrast to a recent report showing no change in SERCA2a expression in human R14Δ-PLN patient hearts [87]. Thus, our work adds to the growing, yet contradictory body of work investigating SERCA2a expression in R14Δ-PLN.

**Table 2:** Gene ontology analysis of proteins with reduced expression in 21wk-old R14^Δ/+^ mouse hearts.

**Proteins depleted in R14**^**Δ/+**^ **mice**			
**GO cellular component**	**Fold enriched**	**FDR**	**Examples**
SAM complex	17.70	9.01E-03	Samm50, Mtx2, Dnajc11
mitochondrial respiratory chain complex I	8.62	6.25E-09	Ndufa1, Ndufa3, Ndufb3, Ndufs6, Ndufc2, Mtnd4, Ndufab1
mitochondrial respiratory chain complex IV	6.64	4.11E-02	Cox7b, mt-Co3, Cox5b, Cox7a2l, Cox7a1, Cox4i1
peroxisome	2.88	5.07E-03	Vimentin, Mul1, Pex19
**Proteins enriched in R14**^**Δ/+**^ **mice**			
**GO cellular component**	**Fold enriched**	**FDR**	**Examples**
*No statistically enriched components detected*			

Protein expression measured by quantitative proteomics (DIA-MS, N = 4). Mitochondrial components highlighted in orange. Gene Ontology performed by Panther v. 18 [68]. Statistical significance determined based on corrected false discovery rate (FDR) below 5% (0.05). Corresponding volcano plots in Fig 8.

### 2.6. Intercalated disk supercomplexes are altered in 28wk-old R14^Δ/+^ mice

In addition to mitochondrial supercomplexes, gene ontology analysis of our Cohort #1 PERCOM hits identified desmosomes as another potentially impacted structure (Table 1). Desmosomes are an important component of the cardiac intercalated disk (ICD), which mediates mechanical and electrical coupling between adjacent cardiomyocytes, as well as cell contacts with fibroblasts and macrophages [88–92]. Within ICDs, mechanical coupling is mediated by the aforementioned desmosomes containing cadherins such as desmoglein-2 (Dsg2), desmoplakin (Dsp) and plakoglobin (Jup), alongside adherens junctions composed of classical cadherin (Cdh2) and catenin-α/β [88]. In direct vicinity, electrical coupling is mediated by gap junctions composed of connexion hemichannels, of which connexin-43 (Cx43) is the major ventricular isoform [88]. Mutations to proteins at the desmosome [93–95], adherens junction [96–99] or gap junction [100, 101] are associated with conduction slowing, ACM and DCM.

**Fig 9:**
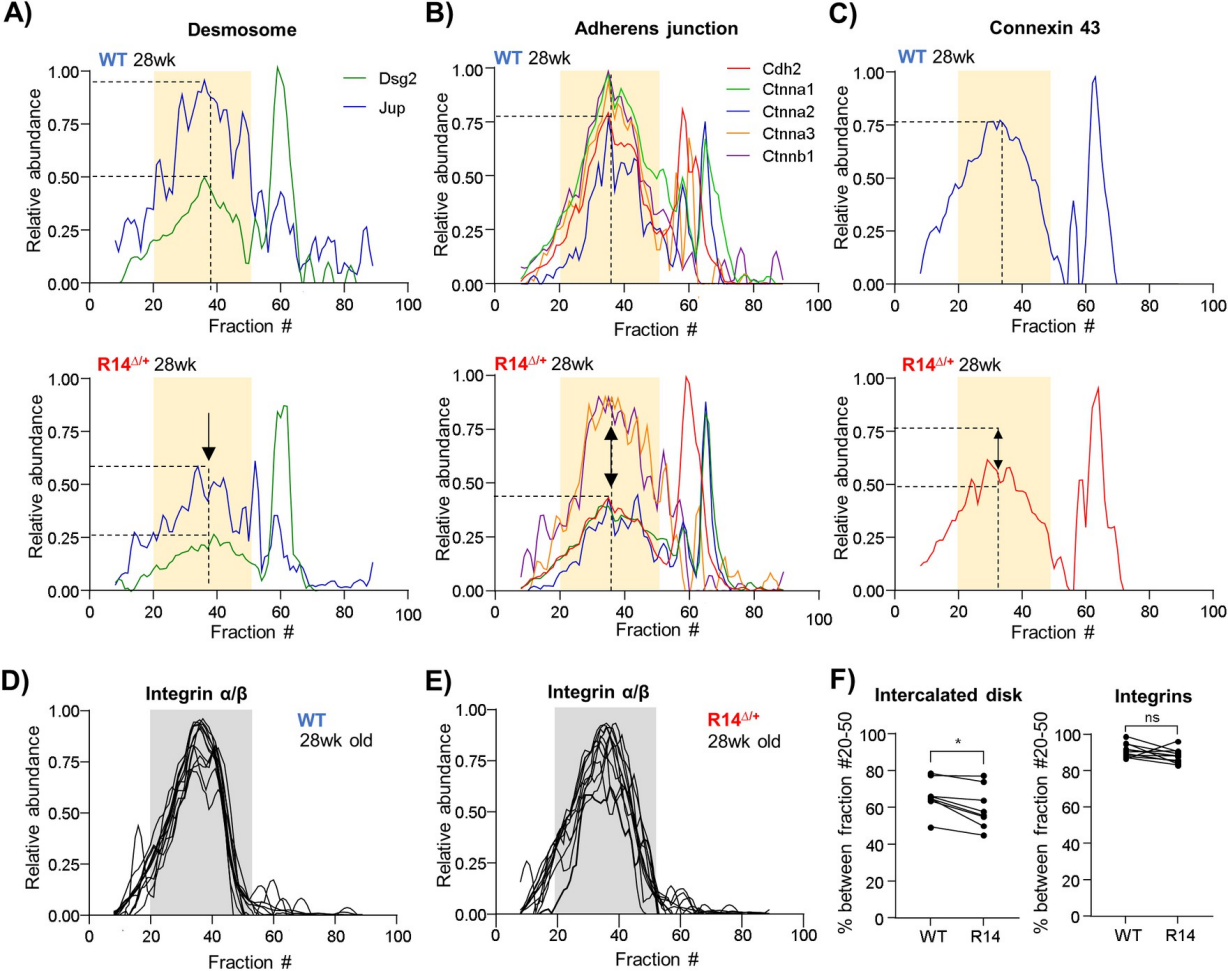
Alterations to intercalated-disk components in 28wk-old R14^Δ/+^- mice. A-C) Elution profiles for structural proteins of the desmosome (A), adherens junction (B) and gap junction (C) from Cohort #1 CP dataset (28wk old ♂ WT vs. R14^Δ/+^ mouse LV tissue, N = 1, Fig 2B). All components coelute as a high-MW peak centered on fraction 38 (vertical dashed line). The distribution of a subset of proteins within this peak is reduced in R14^Δ/+^- mice, as indicated by dashed horizontal lines and arrows. D-E) Elution profiles of Integrin α/β components. Full list of protein IDs in Table 3. F) Fraction of detected ICD and Integrin proteins eluting within MW-fraction 20–50, indicated with yellow and gray underlay, respectively, and presented as in Fig 6. Significance determined via mixed one-way ANOVA with Šidák corrections for multiple comparison (*p < 0.05, ns = no significant difference). All elution profiles subject to curve smoothing as in Fig 2.

The impact of R14Δ-PLN on cardiac desmosomes was evaluated by comparing elution profiles from our Cohort #1 CP dataset. Numerous desmosomal proteins were identified by PERCOM (Table 3 and S3 Table); however, only a subset (Dsg2, Jup) was detected as high-MW complexes (Fig 9A). Nonetheless, both of these proteins were shifted towards low-MW fractions in R14^Δ/+^ mice (Fig 9A). We then evaluated the integrity of the adherens and gap junction complexes, where we found a similar shift towards lower-MW fractions (Fig 9B and 9C). In contrast, the elution profiles of α/β integrin, a family of unrelated cell-surface adhesion proteins [102] were unchanged (Fig 9D and 9E). Quantification of apparent high-MW ICD peaks was performed as described above and confirmed these observations (Fig 9F).

**Table 3:** Elution profiles from Cohort #1 CP dataset (28wk-old WT vs. R14^Δ/+^) shown in this work.

Cellular component	List of proteins	Corresponding figure
26s Proteasome	Psmc1, Psmc2, Psmc3, Psmc4, Psmc5, Psmc6, Psmd1, Psmd11, Psmd12, Psmd13, Psmd14, Psmd2, Psmd3, Psmd8	Fig 2C
EMC	Emc1, Emc2, Emc3, Emc4, Emc6, Emc7	Fig 2D
Na^+^/K^+^-ATPase	Atp1a1, Atp1a2, Atp1a4, Atp1al2, Atp1b1, Atp4b, Atp1b2, Atp1b3	Fig 2E
Cardiac troponin	Tnnc1, Tnni3, Tnnt2	Fig 2F
Mitochondrial complex CI	Ndufa10, Ndufa11, Ndufa12, Ndufa2, Ndufa3, Ndufa5, Ndufa6, Ndufa8, Ndufa9, Ndufb1, Ndufb10, Ndufb11, Ndufb2, Ndufb3, Ndufb4, Ndufb5, Ndufb6, Ndufb8, Ndufs1, Ndufs2, Ndufs3, Ndufs4, Ndufs5, Ndufs6, Ndufs7, Ndufs8, Ndufv1, Ndufv3	Fig 6A. Text color mirrors that shown in corresponding figure.
Mitochondrial complex CIII	Uqcr10, Uqcr11, Uqcrb, Uqcrc1, Uqcrc2, Uqcrfs1, Uqcrh, Uqcrq, Cyc1, Mt-Cyb	Fig 6B
Mitochondrial complex CV	Atp5c1, Atp5c1, Atp5a1, Atp5d, Atp5e, Atp5g1, Atp5me, Atp5mf, Atp5mg, Atp5mk, Atp5md, Atp5mpl, Atp5pb, Atp5pd, Atp5pf, Atp5po, Mtatp8	Fig 6E
Mitochondrial complex CII	Sdha, Sdhb, Sdhc, Sdhd	Fig 6D
Mitochondrial complex CIV	Cox14, Cox4i1, Cox5a, Cox5b, Cox6a2, Cox6b1, Cox6b, Cox6c, Cox7a1, Cox7a2, Cox7b, Cox7c, Mtco1, Mtco2, Mtco3	Fig 6C
Integrins	Itga2b, Itga3, Itga5, Itga6, Itga7, Itga9, Itgav, Itgb1, Itgb3, Itgb5	Fig 9D–9E

PERCOM hits indicated with coloured text. Only proteins with identities not explicitly listed in the corresponding figure legend are shown here

Overall, these results suggest that intercalated disk architecture may be altered at an early stage of R14Δ-PLN cardiomyopathy. This may contribute to the observed electrophysiological abnormalities such as a reduced conduction velocity observed in R14^Δ/+^ mouse hearts [7, 41] and increased arrhythmia risk in both transgenic mice [7, 41, 103] and human patients [3].

### 2.7. Establishing biological reproducibility of key supercomplex changes in a second cohort of juvenile 9wk-old R14^Δ/+^ mice

Thus far, our observations have come from a single cohort containing one ♂ 28wk-old WT and one ♂ R14Δ^/+^ mouse (Cohort #1, Fig 2B). In order to support biological reproducibility and generalization of these findings, and also to determine whether these alterations occur at even younger ages, we performed an additional SEC-MS complexome profiling experiment on a second cohort of ♂ 9wk-old WT and R14^Δ/+^ mice (Cohort #2, N = 1). Mice reach sexual maturity between 8 to 12 weeks of age, equivalent to ~11.5 years in humans [104]; therefore, this 9wk-old cohort could be analogous in age to early-teenage R14Δ-PLN carriers and will be referred to as “juvenile”.

#### 2.7.1. Fractionation of cardiac membrane protein complexes by size-exclusion chromatography is reproducible within and between experimental cohorts

**Fig 10:**
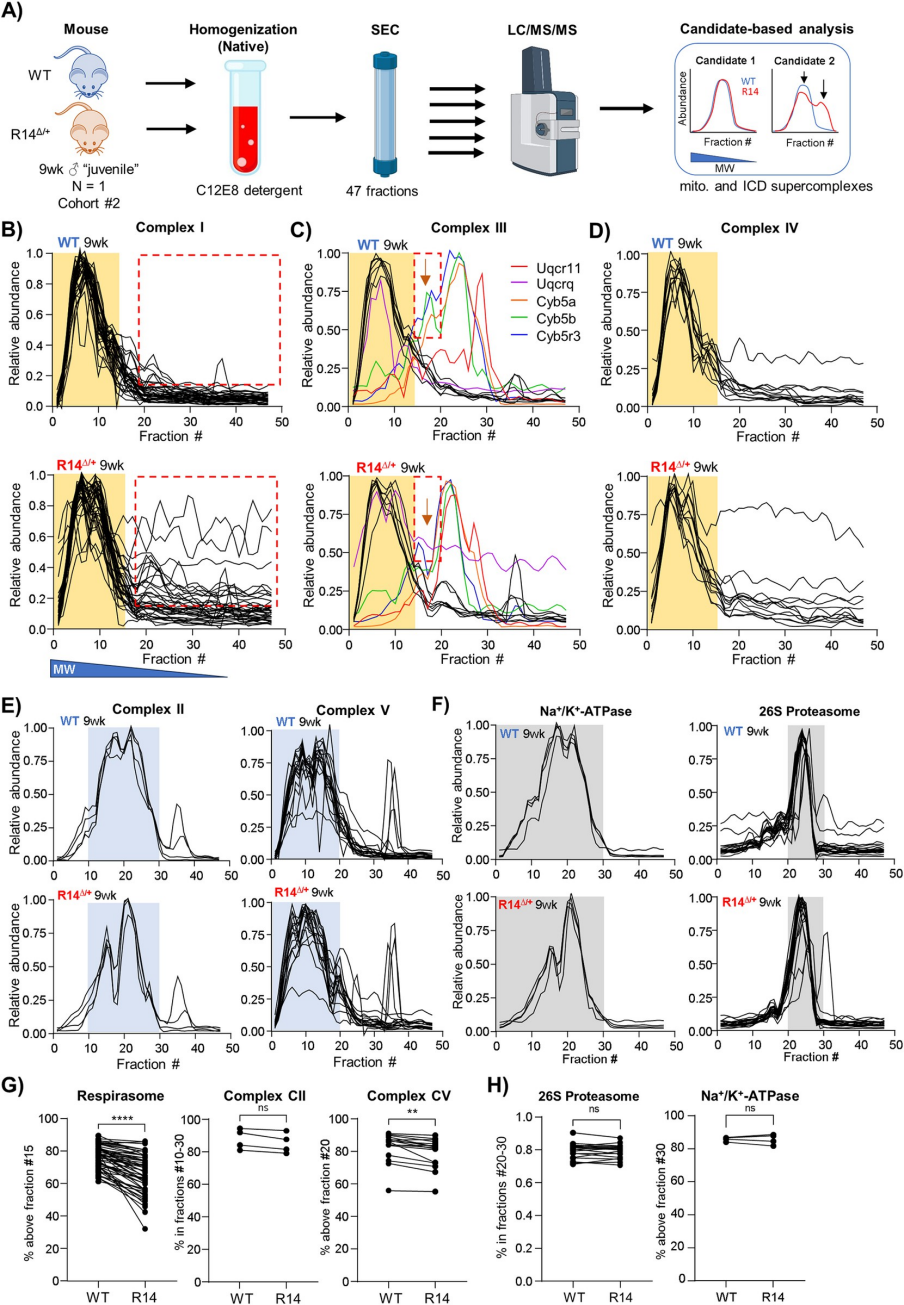
Mitochondrial respirasome supercomplexes alterations reproducibly detected in a second cohort comprising 9wk-old juvenile R14^Δ/+^ mice. A) SEC-MS workflow for Cohort #2 9wk-old mice. LV tissues was dissected from sibling-matched 9wk-old ♂ WT and R14^Δ/+^ mice (N = 1: a single WT and a single R14^Δ/+^). Membrane fractions were enriched by differential centrifugation, solubilized in C12E8 (0.5 mg/mg protein) and separated into 47 fractions by SEC. Each fraction was then subjected to MS-based proteomics analysis. Elution profiles of candidate proteins and protein-complexes were compared. B-D) Elution profiles of respirasome components from 9wk-old WT and R14^Δ/+^ mice. Each trace represents the elution profile of a single protein. Proteins with altered elution profiles indicated by various colors. Respiratory supercomplex (RSC) containing CI, CIII and CIV occupies a high-MW peak between fractions 0 and 15 (indicated with yellow underlay). Areas of notable differences highlighted by red dashed box. E) Elution profiles of mitochondrial complexes CII and CIV, which are not part of the RSC, are unaltered. Both complexes form intermediate-MW peaks (blue) distinct in both shape and apparent MW from the RSC-peak shown in B-D). F) Non-mitochondrial protein complexes are unaltered, supporting technical reproducibility of our workflow. G) Quantification of apparent RSC supercomplex integrity as in Figs 2 and 6. Significance determined via mixed one-way ANOVA with Šidák corrections for multiple comparison. ****p < 0.0001, ** p < 0.01, ns = no significant difference. Full list of protein IDs in Table 4. All elution profiles subject to curve smoothing as in Fig 2.

Cohort #2 consists of a single ♂ 9wk-old WT-mouse and a single sibling-matched ♂ R14^Δ/+^ mouse (N = 1, Fig 10A). SEC-MS complexome profiling was performed using the same workflows used for Cohort #1. As our primary objective was to confirm alterations in PERCOM-derived hits rather than identification of new proteins of interest, data analysis was limited to candidate-based manual workflows. In addition, the number of collected fractions was reduced from 89 to 47 (Fig 10A). Here, 2139 proteins were detected across both Cohort #2 animals. SEC chromatogram and MW-calibration curves are shown in S7 Fig, and elution profiles for all detect proteins in S5 and S6 Tables.

**Table 4:** Protein elution profiles from Cohort #2 CP dataset (9wk-old WT vs. R14^Δ/+^) shown in this work.

Cellular component	List of proteins	Corresponding figure
Mitochondrial complex CI	Ndufa1, Ndufa10, Ndufa11, Ndufa12, Ndufa13, Ndufa2, Ndufa3, Ndufa5, Ndufa6, Ndufa7, Ndufa8, Ndufa9, Ndufb1, Ndufb10, Ndufb11, Ndufb2, Ndufb3, Ndufb4, Ndufb5, Ndufb6, Ndufb7, Ndufb8, Ndufb9, Ndufc2, Ndufs1, Ndufs2, Ndufs3, Ndufs4, Ndufs5, Ndufs6, Ndufs7, Ndufs8, Ndufv1, Ndufv2, Ndufv3	Fig 10B
Mitochondrial complex CIII	Uqcr10, Uqcr11, Uqcrb, Uqcrc1, Uqcrc2, Uqcrfs1, Uqcrh, Uqcrq, Cyc1, Cyb5a, Cyb5b, Cyb5r3	Fig 10C. Text colour mirrors that shown in corresponding figure.
Mitochondrial complex CV	Atp5c1, Atp5a1, Atp5b, Atp5d, Atp5e, Atp5g2, Atp5me, Atp5mf, Atp5mg, Atp5mj, Atp5mk, Atp5pb, Atp5pd, Atp5pf, Atp5po, Mtatp8	Fig 10E
Mitochondrial complex CII	Sdha, Sdhb, Sdhc, Sdhd	Fig 10E
Mitochondrial complex CIV	Cox4a, Cox5a, Cox5b, Cox6a2, Cox6b, Cox6c, Cox7a1, Cox7a2, Cox7b, Cox7c, Mtco2, Mtco3	Fig 10D
Na^+^/K^+^-ATPase	Atp1a1, Atp1a2, Atp4b, Atp1b3	Fig 10F
26s Proteasome	Psmc1, Psmc2, Psmc3, Psmc4, Psmc5, Psmc6, Psmd1, Psmd11, Psmd12, Psmd13, Psmd14, Psmd2, Psmd3, Psmd4, Psmd5, Psmd6, Psmd7, Psmd8, Psmd9	Fig 10F
Integrins	Itga6, Itga7, Itgb1, Itbg2	Fig 11F and 11G

**Fig 11:**
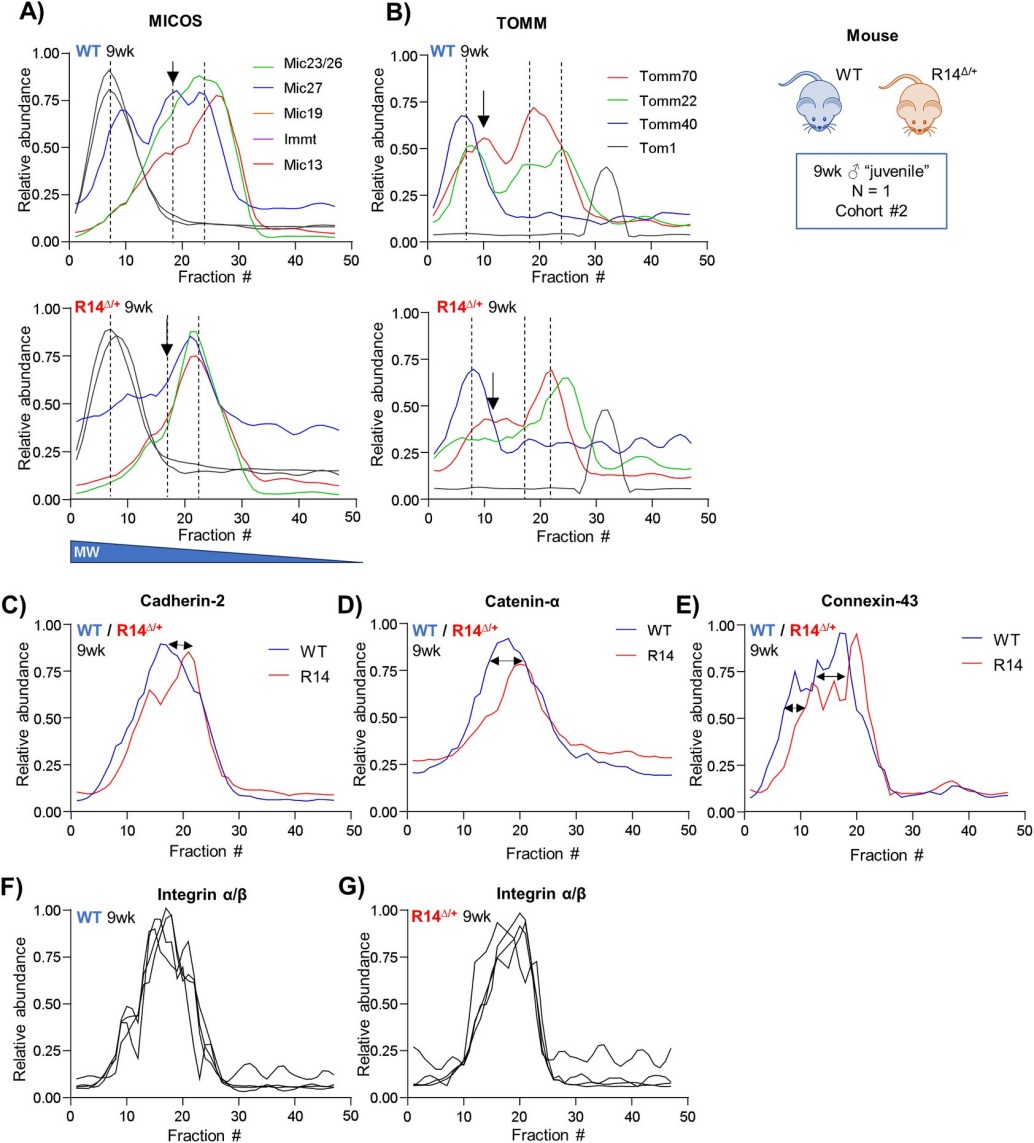
A putative MICOS/SAM/TOM supercomplex and intercalated disk assemblies are altered in a second cohort of 9wk-old juvenile R14^Δ/+^-mice. A-B) Elution profiles of MICOS and TOM proteins detected in juvenile 9wk-old WT and R14^Δ/+^ mice (Cohort #2, ♂, LV tissue, N = 1, Fig 10A). Co-eluting peaks indicated with dotted lines. SAM50 complex components were not detected in this experiment. Areas of notable difference indicated by arrows. C-E) Elution profiles of all detected adherens proteins (cadherin-2, catenin-A) and gap junction protein connexin-43 from juvenile 9wk-old WT and R14^Δ/+^ mice. Elution profiles of all proteins shifted towards lower-MW fractions in R14^Δ/+^ (arrows). F-G) Elution profiles of integrin α/β are not altered in 9 wk-old R14^Δ/+^ mice. Full list of protein IDs in Table 4. All elution profiles subject to curve smoothing as in Fig 2.

Only proteins with identities not explicitly listed in the corresponding figure legend are shown here. Corresponds to elution profiles shown in Figs 10 and 11.

In order to support technical and biological reproducibility both within and between our two cohorts, we performed a heatmap analysis of all CP datasets. As discussed above, this analysis is an established method for evaluating reproducibility of complexome-profiling experiments [60, 61]. We selected a panel of proteins which met the following criteria: A) were detected across all datasets (Cohorts #1 and #2, WT and R14^Δ/+^) and B) were part of established protein complexes defined within the CORUM 4.0 database (as evaluated using the mCP R-script [33]). mCP identified 30 CORUM-defined complexes common across all datasets (S8 Fig) comprising 125 proteins. Heatmap analysis of these proteins supported technical and biological reproducibility both within and between our two cohorts (S8 Fig).

#### 2.7.2. Mitochondrial supercomplex alterations in 9wk-old R14^Δ/+^ mouse hearts

Having established a baseline degree of biological/technical reproducibility both between and within experimental cohorts, we then asked whether the observed changes in the mitochondrial respirasome and MICOS/TOM/AM supercomplex observed in our Cohort #1 dataset from adult 28wk-old animals could be replicated in our Cohort #2 dataset from juvenile 9wk-old animals. The elution profiles of respiratory complexes CI, CIII and CIV were compared (Fig 10B–10D). Once again, we found similar alterations in the elution profiles for CI and CIII. Mitochondrial complexes CII and CV, and unrelated protein complexes such as the 26S proteasome and Na^+^/K^+^-ATPase were unchanged (Fig 10E and 10F). This was confirmed by quantification of the percent of each protein lying within the identified high-MW peak as in Figs 2 and 6 (Fig 10G and 10H).

Changes in the putative MICOS/TOM/SAM supercomplex and IMMP complex in our juvenile 9wk-old animals were similarly assessed. While we were unable to detect components of the SAM and IMMP complexes in our more limited Cohort #2 dataset, we did find changes to the elution profiles of MICOS and TOM components (Fig 11A and 11B). Taken together, these results suggest that functional and protein-level alterations at the mitochondria represent extremely early, potentially pathological changes occurring in juvenile (9wk-old) animals.

**2.7.3. Intercalated disk alterations in 9wk-old R14**^**Δ/+**^ **mouse hearts.** Lastly, we evaluated the integrity of intercalated disk complexes in juvenile (9wk-old) Cohort #2 mice. We were unable to detect desmosomal proteins in high-MW fractions; however, all detected adherens proteins cadherin-2 (Cdh2) and catenin-α1 (Ctnna1), as well as Cx43 showed altered elution profiles (Fig 11C–11E). Integrins once again served as negative controls and were unaffected (Fig 11F and 11G).

## 3. Discussion

### 3.1. Molecular-level mitochondrial alterations are a hallmark of early-stage R14Δ-PLN cardiomyopathy

Here, we present three lines of evidence demonstrating mitochondrial alterations as a hallmark of early-stage R14Δ-PLN cardiomyopathy in presymptomatic (9-28wk-old) R14^Δ/+^ mice: altered elution profiles of both the RSC and a putative MICOS/SAM/TOM supercomplex (via complexome profiling), reduced expression of RSC and SAM component proteins (via MS-based proteomics analysis) and reduced maximal OCR (via Seahorse). Previous work has shown reduced mitochondrial function in patient-derived hiPSC-CM models [53] and, only recently, in presymptomatic R14^Δ/+^ mice [14]. Thus, our results are not only among the first reports of mitochondrial alterations as an early-disease event in presymptomatic animals, but also provide the first direct evidence of changes at the molecular/protein level. Observed changes to mitochondrial protein elution profiles were relatively subtle, suggesting minor shifts towards smaller sub-assemblies rather than widespread supercomplex disruption. This is consistent with recent work showing no significant change to mitochondrial membrane potential and cytosolic reactive oxygen species (ROS) content in 8-12wk-old R14^Δ/+^ mice [14] and the overall lack of significant cardiomyopathy in these animals [7, 14], which together paint a picture of subtle changes to mitochondrial organization and function.

#### 3.1.1. Observed molecular changes may be functionally and causally related

Our combined CP and proteomics data identified changes in select protein complexes (IMMP, MICOS, SAM and TOM) and the expression of individual proteins (COX7A2L and NDUFAB1) that may influence mitochondrial organization and impede the assembly of mitochondrial complexes CI/III/IV into efficient respiratory supercomplexes [65, 74, 75, 81–84]. Furthermore, loss of COX7A2L and NDUFAB1 expression has been associated with respiratory supercomplex disruption, reduced mitochondrial function and increased ROS production in cardiac systems [65, 85]. Thus, it seems reasonable to speculate that changes in the IMMP and MICOS/SAM/TOM complexes and/or COX7A2L/NDUFAB1 protein expression lie upstream of RSC disruption and, ultimately, loss of mitochondrial function. Causality will ultimately need to be explored in future work (potentially by combining genetic ablation of key MICOS/SAM/TOM machinery or COX7A2L/NDUFAB1 alongside R14Δ-PLN knockin expression).

#### 3.1.2. Possible mechanisms underlying mitochondrial dysfunction

**Fig 12:**
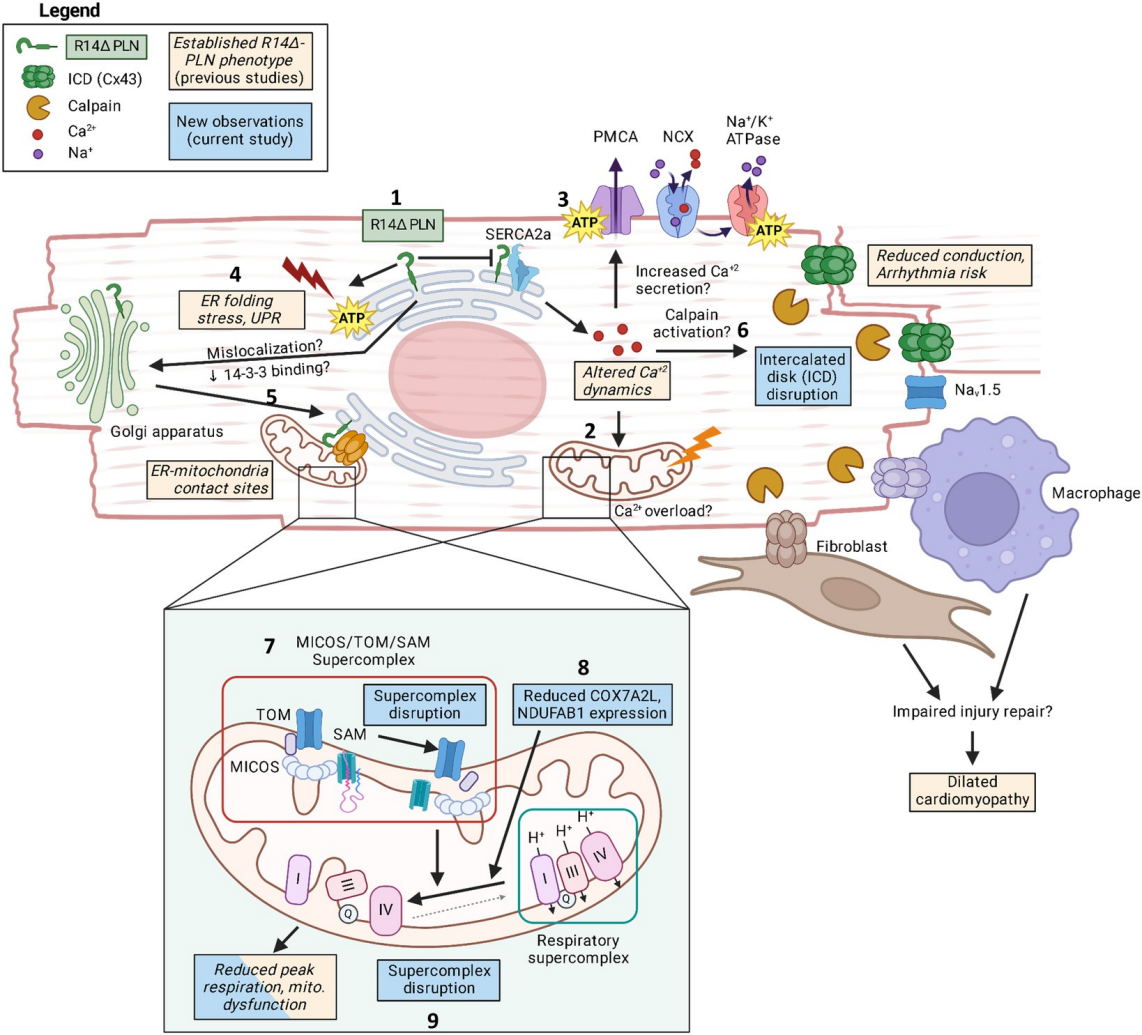
Hypothetical cellular model of observed molecular changes. Previous work has associated R14Δ-PLN **(1)** with a complex phenotype at both the cellular and whole-organism level. These include: altered Ca^2+^-dynamics commonly attributed to SERCA2a superinhibition, ER folding stress and upregulated unfolded protein response (UPR), impaired ER-mitochondrial contact sites, reduced mitochondrial function, reduced conduction velocity, and arrhythmogenic and/or dilated cardiomyopathy (yellow text boxes). In our present study, we identify additional changes at the mitochondria, including alterations to a putative MICOS/TOM/SAM supercomplex, reduced expression of mitochondrial respiratory chain assembly factors COX7A2L and NDUFAB1, alterations to the CI/III/IV respiratory supercomplex (RSC) and reduced peak mitochondrial output (blue text boxes). We propose several hypothetical pathways linking R14Δ-PLN to mitochondrial dysfunction: abnormal Ca^2+^-dynamics may lead to mitochondrial Ca^2+^-overload **(2)**, increased ATP-dependent Ca^2+^-secretion via plasma-membrane transporters may result in increased ATP demand and further mitochondrial stress **(3)**, ER-folding stress, UPR activation, and upregulation of ATP-dependent proteostasis pathways may also contribute to increased energy demand and mitochondrial stress **(4)** and, lastly, loss of diarginine ER-retention signal and/or binding of phospho-adaptor protein 14-3-3 may result in aberrant trafficking of R14Δ-PLN to post-ER compartments, where it may interfere with ER-mitochondrial contact sites **(5).** In addition to alterations at the mitochondria, we also observed changes at the intercalated disk (ICD, **6**). We hypothesize that abnormal Ca^2+^-dynamics may result in aberrant Calpain activity and excessive cleavage of intercalated disk (ICD) components (Connexin-43, Cx43 illustrated as example). Intercalated disks mediate critical contacts with neighboring cardiomyocytes, macrophages and fibroblasts, and are vital for conduction propagation and injury repair, respectively. We speculate that loss of this function may contribute to the impaired conduction phenotype, arrhythmogenic and dilated cardiomyopathy. ***Inset***: at the mitochondria, we describe a putative MICOS/TOM/SAM supercomplex **(7)** that is likely involved in import/insertion of mitochondrial proteins, organization of mitochondrial membrane topology, and stabilization of respiratory supercomplexes containing complexes CI/CIII/CIV. We hypothesize that alterations to this supercomplex, in addition to loss of expression of assembly factors NDUFAB1 and COX7A2L **(8)** may lead to a partial disruption of the respiratory supercomplex and reduced respiratory efficiency **(9)**.

How R14Δ-PLN (an integral SER-membrane protein) can exert influence in the mitochondria remains an open question. While a definitive answer lies beyond the scope of this work, we would nonetheless like to put forth four speculative hypotheses for discussion and potential future investigation and experimental validation (Fig 12).

1. R14Δ-PLN is associated with various Ca^2+^-handling defects, including a hyperdynamic phenotype [14] and increased diastolic [Ca^2+^]_i_ [86]. Altered Ca^2+^ dynamics may lead to mitochondrial Ca^2+^-overload resulting in damage to mitochondrial supercomplexes [105]. A similar mechanism has been implicated as a key determinant of heart failure pathology [106]. The recent observation that acute reduction of cytosolic or SER [Ca^2+^] results in a worsening rather than an improvement in mitochondrial function in R14^Δ/+^ mouse ventricular myocytes, as well as failure to detect increased ROS generation in R14^Δ/+^ mouse ventricular myocytes at rest (a hallmark of Ca^2+^-overload [105, 107]) would appear to argue against this [14].
2. Impaired Ca^2+^-reuptake may require compensation via increased Ca^2+^ secretion by the plasma membrane Ca^2+^-ATPase (PMCA) and/or the Na^+^/Ca^2+^-exchanger (NCX, with increased Na^+^ influx being countered by increased Na^+^/K^+^-ATPase activity) [108], resulting in increased ATP demand and metabolic imbalance. In addition, increased UPR activity [13] and protein aggregation [7, 12] may also increase ATP demand [109, 110]
3. R14Δ-PLN is associated with increased activation of unfolded protein response (UPR) pathways, ER stress [13] and formation of PLN-positive perinuclear aggregates [7, 12]. Proteostatic imbalance may further increase cellular demand for ATP (e.g. via increased activity of ATP dependent chaperones and proteasomal degradation) [109, 110], further taxing the mitochondria.
4. Mislocalization of a subpopulation of R14Δ-PLN to post-ER compartments, previously observed in both HEK293 cells and transgenic mouse models [111, 112], may contribute to mitochondrial dysfunction. The R14Δ mutation disrupts a R13/R14 diarginine motif that may act as an ER-retention signal [113, 114] and 14-3-3 binding site [115]; this may result in the mislocalization of a subpopulation of R14Δ-PLN to peripheral post-Golgi cellular compartments [113, 116]. Our own super-resolution microscopy analysis on isolated mouse ventricular cardiomyocytes did not reveal any gross redistribution or mislocalization of PLN; however, it is possible that only a small subpopulation of PLN is mislocalized in our R14^Δ/+^ mouse model, which may evade detection by imaging techniques. Interestingly, our complexome profiling data shows that PLN co-elutes with a subset of ER-mitochondrial tethering proteins, including Calnexin, BCAP31 and VAPA/B (S9 Fig), suggesting some degree of crosstalk (Fig 12). In addition, the mitochondrial transmembrane protein TMEM 126B was identified as a hit in our PERCOM analysis (S4 Table). TMEM 126B plays a key role in the assembly of mitochondrial complex CI via the Mitochondrial Complex I Assembly (MCIA) complex [29]. TMEM126B showed increased tendency to comigrate with PLN in 28wk-old R14^Δ/+^ mice compared to WT controls (S9 Fig); it was not detected in our 9wk-old cohort dataset. As TMEM126B may span both the inner and outer mitochondrial membranes, this provides a potential direct physical link between PLN and IMM supercomplex integrity.

### 3.2. Intercalated disks may represent a new site of R14Δ-PLN action

Previous reports observed a decrease in conduction velocity in 12–16wk-old R14^Δ/+^ mice hearts [7, 41], which may contribute to the R14Δ-ACM phenotype and is relevant for the 28wk-old mice studied here. Due to the complete absence of fibrosis at this age [7, 11], these conduction abnormalities have been initially attributed to altered Ca^2+^-homeostasis [7]. ICDs mediate electromechanical coupling between adjacent cardiomyocytes, macrophages [89] and fibroblasts [91, 92]. In addition to acting as physical portals, intercalated disks also recruit voltage-gated cation channels such as Na_v_1.5 to propagate depolarization at the gap junction to the rest of the cell [117, 118]. Mutations to intercalated disk proteins such as desmoglein-2, plakophillin-2 (PKP2) and plakoglobin are associated with decreased conduction velocity and ACM [119, 120]. In addition, contact between cardiomyocytes, macrophages and fibroblasts is involved in response to cardiac injury [90, 91], and ICD disruption has also been recently implicated in DCM pathogenesis [121]. Here, we provide preliminary evidence that intercalated disk supercomplexes are altered in R14^Δ/+^ mice, which may contribute to the ACM/DCM phenotype (Fig 12). Further functional and (ultra)structural experiments should be done to establish this as a potential disease mechanism.

Calpain is a peripheral Ca^2+^-dependent protease that may be activated in several cardiac disease conditions. Previous work has shown that aberrant proteolysis of IDC components by Calpain may contribute to myocardial infarction and atrial fibrillation pathology [122–124]. We hypothesize that the hyperdynamic Ca^+2^-phenotype in R14Δ-PLN hearts may trigger Calpain overactivation and ICD degradation (Fig 12). We implicated a similar mechanism in the degradation of the key SER-PM membrane tethering protein Junctophilin-2 (JPH2) in other cardiac disease [125–127]. Indeed, due to its established Calpain sensitivity, JPH2 may be another substrate of interest in R14Δ-PLN.

### 3.3. Mitochondrial and intercalated disk supercomplexes represent potential therapeutic targets for early intervention

A critical component of our work is the observation of disrupted ICD and mitochondrial supercomplexes in a second independent cohort of juvenile (9wk-old) R14^Δ/+^ mice (corresponding to ~11-12-year-old human, [104], Fig 10A), significantly prior to the onset of cardiomyopathy at 18 months of age in mice [7, 11]. Targeting these pathways at a very young age may represent an effective therapeutic strategy that can be delivered prior to the onset of clinical symptoms. Recent work has shown AAV-mediated overexpression of mitochondrial complex CI core subunit S1 (Ndufs1) and assembly factor Ndufab1 to be cardioprotective against ischemia-reperfusion injury in mice [65, 128]. These gene therapy approaches may be of use in treating R14Δ-CM. In particular, Ndufab1 overexpression enhances assembly of the RSC [65], which is a specific alteration seen in R14^Δ/+^ mice. Alternatively, several mitochondrial-targeting drugs such as the mitochondria-targeted antioxidant MitoQ [129, 130] and Elamipretide (a small molecule inhibitor of ROS formation) [131] have been trialed for a wide range of diseases including Alzheimer’s [132] and Parkinson’s disease [132]. While preclinical and clinical testing has yielded mixed results [133–137], the potential application to R14Δ-PLN remains unexplored. In particular, the very early onset of mitochondrial alterations may make preventative treatment particularly effective (conversely, the inability to treat prior to onset of clinical symptoms may have limited the apparent effectiveness of these compounds in prior clinical trials) [135]. Therapeutic approaches targeting the ICD are less established. Should increase and/or aberrant Calpain activity be detected in R14^Δ/+^-PLN hearts (potentially leading to degradation of ICD components), testing of Calpain inhibitors may be warranted.

### 3.4. Application of SEC-DIA-MS based complexome profiling workflows in cardiac samples

Previous complexome profiling studies on cardiac tissue employed digitonin-based solubilization of membrane and/or mitochondrial proteins followed by BNE-based fractionation and MS-based proteomics measurement [22, 42]. We made several critical improvements to this data acquisition workflow: **1)** *Choice of detergent*. C12E8 was found to be superior to digitonin for the solubilization of cardiac membrane protein complexes (S1 and S2 Figs). In addition, C12E8 is a synthetic detergent that is commercially available at high purity and homogeneity, whereas digitonin represents a complex mix of glycosides isolated from *Digitalis purpurea*, resulting in both lower purity and increased lot-to-lot variability [138]. **2)** *High MW range SEC-based fractionation*. This study is, to our knowledge, the first application of SEC-based fractionation to cardiac samples. Compared to BNE-PAGE, SEC offers potentially-enhanced resolution at very high (here, up to 7.5 MDa) MW ranges and enhanced reproducibility [45]. **3)** *Quantitative DIA-MS analysis using an empirical spectral library*. Previous work relied upon *in-silico* tryptic digests to generate peptide libraries for DIA data extraction. Here, we generated a dedicated spectral library specific for adult C57BL/6J mouse ventricular tissue in order to improve bottom-up proteomic depth of analysis [139]. More generally, DIA-MS has been demonstrated to increase both the speed and the reproducibility of CP experiments. Taken together, our SEC-DIA-MS workflow offers significant improvements for complexome profiling analysis in cardiac samples. This is illustrated by our ability to detect not only routinely-studied mitochondrial complexes and supercomplexes, but also cardiac-specific structures such as the SER Ca^2+^-handling supercomplex, voltage-gated Ca^2+^ channel and K^+^- channel oligomers, and intercalated disk components.

### 3.5. PERCOM: An accessible and unbiased protein-centric data analysis workflow able to detect subtle changes in specialized tissue-specific supercomplexes

In addition to the improved data acquisition strategy, we also developed PERCOM as a novel data analysis workflow. PERCOM bypasses several issues associated with using curated protein complex databases for ground-truth (underrepresentation of tissue- or cell-type-specific structures, underrepresentation of higher-order supercomplexes with fluid composition, incomplete coverage of the human proteome) and can, furthermore, be implemented using basic spreadsheet software, making it broadly accessible to the scientific public.

It should also be noted that PERCOM was able to identify proteins with subtly-altered elution profiles in a presymptomatic heterozygous animal model [7, 11]. This highlights the sensitivity of our workflow, and demonstrates its suitability for use in disease models with no/mild phenotypes where only subtle alterations might be expected. There is a growing appreciation that many critical functions in excitable cell types, such as Ca^+2^-signalling, excitation propagation and membrane contacts, occur with nanoscale resolution via specialized supercomplexes or membrane nanodomains [22, 140]. We believe that our SEC-DIA-MS CP workflow coupled with PERCOM represents important tools for studying higher-order protein complexes underlying these nanoscale functions in specialized cell-types, and identifying alterations in mild/asymptotic disease models.

### 3.6. Study limitations

#### 3.6.1. Establishing biological reproducibility from limited sample size

This study presents two CP datasets: one from 28wk-old ♂ mice (Cohort #1, Figs 1–9), and one from 9wk-old ♂ mice (Cohort #2, Figs 10 and 11). The experimental cohort for each experiment was a single R14^Δ/+^ animal and a sibling-matched WT-control (N = 1). Due to the large number of MS-based proteomics measurements required per animal (47 or 89 fractions per sample, each measured in technical duplicate), biological replicate measurements were cost- and time-prohibitive. The same cost issue also precluded experiments on female animals. Nonetheless, our core findings were observed in both Cohort #1 and Cohort #2 datasets, despite the differences in both age and cohort, supporting biological reproducibility of our results. Lastly, while our observations at the mitochondria are supported by multiple lines of evidence (complexome profiling, global proteomics, Seahorse), as well as a body of previous work in both hiPSC-CMs [53] and transgenic mice models [14], our observations at the intercalated disk are admittedly limited to a single readout with no prior art. We believe further research into this topic, including orthogonal functional and (ultra)structural validation of the intercalated disk, to be an important direction for future work.

#### 3.6.2. Determining the biological impact of complexome-level changes

In this study, we identify changes to several functionally-critical protein complexes and supercomplexes comprising the cardiac intercalated disk and involved in mitochondrial organization and function. These complexome-level changes are functionally linked to known disease phenotypes (e.g. impaired cardiac conduction and increased arrhythmia risk) or validated cellular dysfunctions (e.g. mitochondrial dysfunction). It is therefore plausible that complexome changes lead to biological impact. Nonetheless, more detailed experiments, such as genetic ablation of key supercomplex components combined with R14Δ-PLN knockin, are required to definitively establish causality. Our proteomics data provides two potential proteins of interest; these candidates (COX7A2L and NDUFAB1) play established roles in RSC assembly, and could be candidate targets to interrogate.

## 4. Conclusions

Here, we describe the development of improved data acquisition and analysis workflows for the profiling of high-MW assemblies in cardiac tissues. Using these workflows, we identified alterations in the elution profiles of several key mitochondrial and ICD components. These findings could be observed in presymptomatic R14^Δ/+^ mice as young as 9 weeks of age. Cardiomyocytes have a uniquely high demand for ATP due to their constant contractile activity, and are dependent on mechanical and electrical connectivity between adjacent cells to facilitate force transduction and propagation of electrical excitation; thus, our observed alterations of mitochondrial and ICD supercomplexes could have increasingly severe consequences to cardiac function. While previous studies have shown impaired mitochondrial function in hiPSC-CM models [53], our findings are among the first to make this observation in a presymptomatic animal system [14], and to demonstrate alterations at the molecular and protein levels.

## 5. Methods and materials

### 5.1. Animal studies

Generation and characterization of the R14Δ-PLN knockin mouse line was described previously [7]. All experimental cohorts consist of sibling-matched male WT and R14^Δ/+^ mice unless otherwise noted. Animal handling was performed in accordance to directive 2010/63/EU of the European Parliament and in keeping with NIH guidelines. In all cases, mice were used for organ extraction to isolate left ventricular tissues and myocytes. Breeding of R14Δ-PLN mice is covered by animal protocol 21/3698, which was approved by the veterinarian state authority (LAVES, Oldenburg, Germany). Breeding of WT-animals for assay development is covered by institutional animal protocols reviewed and approved by the institutional animal committee of the University Medical Center Göttingen. Animals were housed in the Universitätsmedizin Göttingen Zentrale Tierexperimentelle Einrichtung (ZTE) and colonies are maintained at the Max Planck Institute for Multidisciplinary Sciences (Göttingen). Animals were anesthetized with isofluorane and sacrificed by cervical dislocation following approved protocols. In all cases, all efforts were made to minimize suffering.

### 5.2. Dissection of ventricular tissue and isolation of enriched membrane fractions

Male WT and R14^Δ/+^ mice were anaesthetized with isoflurane and sacrificed by cervical dislocation following approved protocols. Whole hearts were dissected, cannulated at the aorta and profused for 4 minutes with a modified Ca^+2^-free Krebs buffer via Langendorf setup at a flow rate of 4ml/min [141]. Flash-frozen left- and right-ventricular tissues were thawed, minced and resuspended in sucrose homogenization buffer (250mM sucrose, 20mM imidazole, 6mM EDTA, 6mM Tris HCl pH 6.8) supplemented with EDTA-free protease inhibitor cocktail (Roche). Tissues were disrupted using potter homogenizer (2x 25 strokes at 2000rpm), followed by passage through a 27G needle (10x). Homogenates were centrifuged at 1,000g (10 minutes, 4°C) to remove debris, and enriched membrane fractions were pelleted by centrifugation at 100,000g (1h, 4°C). Membrane fractions were solubilized for at-least 30 minutes at 4°C in sucrose homogenization buffer containing either digitonin (6mg/mg protein), Triton X-100 (0.01mg/mg protein), NP-40 (0.5mg/mg protein) or C12E8 (0.5mg/mg protein).

### 5.3. Generation of empirical spectra library and DDA/DIA-MS proteomics analysis

50 μg of enriched membrane fractions were solubilized in either SDS (2%) or C12E8 (0.5 mg/mg protein) and purified by a brief SDS-PAGE run (Nu-PAGE 4–12%, Invitrogen). Lanes were excised, reduced, alkylated, and digested with trypsin as previously described [142]. Tryptic digestion was quenched using 1% trifluoracetic acid and peptides were separated into 12 fractions by basic reverse-phase liquid chromatography (äkta pure, Bruker). Liquid chromatography (LC)-coupled tandem MS/MS analysis was performed using a hybrid timed ion mobility-time of light mass spectrometer (timsTOF Pro 2, Bruker) coupled to a nanoflow chromatography system (Ultimate nanoSRLC, Thermo Fisher Scientific) equipped with a reversed phase-C18 column (Aurora Elite 250x0.075 mm, IonOpticks) and employing a linear 2–37% acetonitrile versus 0.1% formic acid gradient at a flow rate of 250 nl/min. Samples were analyzed on in either DDA mode (one technical replicate) or DIA mode (two technical replicates). DDA analysis was performed in Parallel Accumulation−Serial Fragmentation (PASEF) mode [143] with 10 scans per topN acquisition cycle. Multiple charged precursors were selected based on their position in the *m/z*–ion mobility plane and isolated at a resolution of 2 Th for *m/z* ≤ 700 and to 3 Th for *m/z* > 700 with a target MS/MS ratio of 20,000 arbitrary units. Dynamic exclusion was set to 0.4 min. DIA analysis was performed in diaPASEF mode [144, 145] using a customized 8x2 window acquisition method from *m/z* from 100 to 1,700 and from 1/K_0_ from 0.7–1.5 to include the 2^+^/3^+^/4^+^ population in the *m/z*–ion mobility plane. The collision energy was ramped linearly as a function of the mobility from 59 eV at 1/K_0_ = 1.5 Vscm^−2^ to 20 eV at 1/K_0_ = 0.7 Vscm^−2^. The spectral library was built with Spectronaut software version 16.3 (Biognosys) by matching data against the Uniprot KB mouse reference proteome (01/2021).

### 5.4. BNE-based complexome profiling for detergent selection

Membrane fractions from seven 28wk-old ♂ WT mice were pooled and solubilized in the indicated detergent. 35–50 μg of solubilized membrane fractions were loaded onto pre-cast Blue Native-PAGE gels (3–12% Bis-Tris minigels, Invitrogen) and run using standard protocols [146]. Lanes were cut into 35 equidistant fractions using a custom stainless-steel cutter. All fractions were reduced, alkylated, digested with trypsin and dried by SpeedVac (Thermo) in 96 well microplates as previously described [142]. Fractions were spiked with iRT standard peptides (1/100 diluted, Biognosys) and subject to LC-MS/MS analysis in technical duplicates on a hybrid quadrupole-orbitrap mass spectrometer (Q Exactive, Thermo Fisher Scientific) hyphenated to a nanoflow chromatography system (Easy-nLC 1000, Thermo Fisher Scientific) equipped with a homemade analytical column (C-18aq, 3μm, 200x0.075mm, Dr. Maisch) and precolumn (Reprosil C18aq, 5μm, 20x0.15 mm, Dr. Maisch). Separation was achieved using a 5% to 35% acetonitrile gradient in 0.1% formic acid. Samples were measured in technical duplicate in DIA mode. Briefly, MS1 spectra were collected in the range of 350–1250 *m/z* at 70,000 resolutions (FWHM), a maximum injection time of 50ms and a target AGC of 1 x 10^6^. Subsequently, 11 variable size DIA windows were analyzed using the following settings: default charge 3^+^, resolution 35,000 (FWHM), maximum injection time 115 ms, AGC target 3x 10^6^, fixed first mass 200 *m/z*. Precursor fragmentation was achieved using a stepped Normalized Collision Energy regime of 26/28/30%. Protein concentration in each fraction was measured by UV absorbance at 280nm prior to analysis (S1 Fig). Spectral data were analyzed using Spectronaut software version 16.3.

### 5.5. SEC-based complexome profiling in adult 28wk-old mice

Membrane fractions were isolated from 28wk-old ♂ WT and R14^Δ/+^ mice (N = 1) as described above. Approximately 120 μg of solubilized membrane fractions were fractionated into 89 fractions using an äkta pure chromatography system (Cytiva) equipped with a Bio-SEC-5 1000Å analytical column (300x7.8 mm, Agilent) and a SEC-5 1000 Å guard column (50x4.6 mm, Agilent). Commercial gel filtration standards (Bio-Rad) were employed for molecular weight calibration of the SEC runs. SEC was performed on ice with an isocratic flow rate of 0.5 ml/min for 36.8 minutes using a buffer containing 5 mM Tris-HCl, 1 mM EDTA, 150 mM KCl, 0.005 mg/ml C12E8 solution (pH 7) [46]. Following fractionation, additional Tris-HCL pH 8.8 (20–25 mM) and SDC (0.2% w/v) was added and reduction, alkylation and tryptic digestion using standard protocols [34]. Fractions were spiked with iRT standard peptides (Biognosys) as described above and separated using a linear 12.5 min gradient of 4–32% aqueous acetonitrile versus 0.1% formic acid on a nanoflow chromatography system (Ultimate nanoRSLC, Thermo Fisher Scientific) using a reversed phase-C18 column (PepSep Fifteen, 150x0.150 mm, Bruker) at a flow rate of 850 nl min^-1^. Peptides were measured in technical duplicates using a hybrid timed ion mobility-time of flight mass spectrometer (timsTOF Pro 2, Bruker). MS/MS analysis was done in diaPASEF mode as described above. Protein concentration in each fraction was measured by UV absorbance at 280 nm. The first 8 fractions (#1 to 8) did not contain sufficient protein concentration to justify measurement and were thus discarded from further analysis (S3 Fig). Spectral data were analyzed using Spectronaut software version 16.3.

### 5.6. SEC-based complexome profiling in juvenile 9wk-old mice

Membrane fractions were isolated from 9wk-old ♂ WT and R14^Δ/+^ mice (N = 1) as described above. Approximately 120 μg of solubilized membrane fractions were fractionation into 47 fractions by SEC as described above. Fractions were spiked with iRT standard peptides and analyzed in technical duplicates via LC-MS/MS on a hybrid quadrupole-orbitrap mass spectrometer (Q Exactive, Thermo Fisher Scientific) hyphenated to a nanoflow chromatography systems (Easy-nLC 1000 UHPLC, Thermo Fisher Scientific) as described above. Protein concentration in each fraction was measured by UV absorbance at 280nm (S7 Fig). Analysis of spectral data was performed using Spectronaut software version 16.3 (Biognosys).

### 5.7. Refinement of complexome profiling data and generation of protein abundance matrices

Following spectral analysis, data was arranged into protein abundance matrices, with rows representing individual protein IDs, columns representing fraction number, and cell values containing the detected abundance (Fig 4A). A Pearson’s correlation cutoff of ≥0.6 between technical duplicates was applied to identify and eliminate proteins with poor technical reproducibility, with an exception being made for RyR2 due to its biological importance and a prime candidate for interrogation. In this case, fractions where the technical replicate measurements of protein abundance differed by more than 25% of maximum were discarded from further analysis. Protein abundance values (expressed as peptide counts) were converted into relative abundances (expressed as fraction of the maximal abundance across the fractions).

### 5.8. Quantification of protein complexes defined by the CORUM protein complex database

Detection and quantification of protein complexes defined within the CORUM 4.0 protein complex database was performed using the mCP R-software package [33]. In brief: protein detection was implemented via detecting co-elution of complex components (coelution being measured by Pearson’s correlation coefficient). A ≥0.81 Pearson’s coefficient cutoff was determined using Monte-Carlo simulations (185 simulations) with a target false-discovery rate of 5%.

### 5.9. Calculation of ΔCoM and Pearson’s correlation coefficient

The elution fraction representing the center of distribution (CoM) was calculated using the following equation:

$$CoD=\sum_{x=1}^{n}\left(x(\frac{P_{x}}{\sum_{x=1}^{n}P_{x}})\right)$$

Where x represents elution fraction number, n represents the total number of fractions, and P represents the relative abundance of the protein in that fraction.

Pearson’s coefficient of correlation was calculated using the following equation [147]:

$$r=\frac{\sum_{x=1}^{n}\left(P_{x}^{wt}-\bar{P^{wt}})(P_{x}^{R14}-\bar{P^{R14}}\right)}{\sqrt{\sum_{x=1}^{n}{\left(P_{x}^{wt}-\bar{P^{wt}}\right)}^{2}}\sqrt{\sum_{x=1}^{n}{\left(P_{x}^{R14}-\bar{P^{R14}}\right)}^{2}}}$$

Where P^WT^ and P^R14^ represent the relative abundance of the protein in the indicated elution fraction number in WT and R14^Δ/+^ samples, respectively.

### 5.10. MS-based proteomics analysis

Left-ventricular cardiomyocytes were isolated from 21wk-old ♂ WT and R14^Δ/+^ mice (n = 4) as described previously [141]. Briefly, whole hearts from 28wk-old ♂ WT and R14^Δ/+^ mice were cannulated and perfused with modified Krebs buffer containing 2 mg/ml collagenase (Worthington) for 10 min. Digested heart tissues were dissected using a scalpel and disrupted by gentle pipetting. Myocytes were isolated by differential sedimentation. Left- and right-ventricles were dissected from digested hearts and disrupted by pipetting. Cardiomyocytes were homogenized by pipetting for 5 minutes at 4°C in lysis buffer (0.05% v/v NP40, 50 mM HEPES, 150 mM NaCl, 50 mM NaF, 200 μM Na_3_VO_4_, pH 7.5) supplemented with 1 mM PMSF and protease inhibitor cocktail (Roche). Lysates were then reduced and alkylated with 10 mM DTT and 100 mM of 2-Chloroacetamide (30 min each step at 30°C) and digested with trypsin (1/40 w/w ratio) at 37°C for 14 h. TMT labelling (1 μg reagent per μg peptide, 50 μg peptide per reaction) was performed at 25°C for 30 minutes and quenched with 5% hydroxylamine (15min at 25°C). Desalting and peptide concentration was performed using reversed phase Sep-Pak C18 cartridges. Labelled peptides were fractionated into 12 fractions using basic pH reversed phase liquid chromatography on a reversed phase-C18 column (Hypersil Gold, 150x2.1 mm, Thermo Fisher Scientific) using a 0% - 90% acetonitrile gradient. LC-MS/MS was performed in technical duplicate using an tribrid mass spectrometer (Orbitrap Fusion, Thermo Fisher Scientific) hyphenated to a nanoflow chromatography system (Ultimate nanoRSLC, Thermo Fisher Scientific) equipped with a reversed phase-C18 chromatography column (Reprosil C18aq, 1.9 μm, 280x0.075 mm, Dr. Maisch).

Spectral data were analyzed using MaxQuant software version 1.6 (Max Planck Institute of Biochemistry). Datasets were analyzed against the UniProtKB mouse reference proteome (06/2019, 55485 entries). Under group specific parameters, the TMT labeling search was made with MS3 reporter ion with the 6plex TMT labeling on lysine and N-terminal residue. Reporter mass tolerance was by default set to 0.003 Da and a maximum of two missed cleavages allowed. Variable modifications include protein N-terminal acetylation and methionine oxidation and fixed modifications contain cysteine carbamidomethylation. Peptides and proteins were reduced using a 1% peptide spectrum match (PSM) false discovery rate (FDR) and a 1% protein FDR determined by the decoy database. Only the razor/unique peptides were used for peptide identification and quantitative calculations.

Protein group data were processed by Perseus v1.6.15.0 (Max-Planck-Institute of Biochemistry). Site only, reverse, and contaminant peptides were removed from the dataset and missing values were imputed using a normal distribution (width: 0.3, downshift: 1.8). Invalid values were then excluded. Empty columns were removed. Rows were categorically annotated as wild type and mutant respectively for each reported intensity corrected and the data was transformed with log2(x). For the removal of the batch effect within TMT 6plex, data normalization was done in Perseus using width adjustment quartile in program normalization.

### 5.11. Mitochondrial functional assays

Left- and right-ventricular cardiomyocytes were isolated as described above and seeded onto fibronectin-coated Seahorse XF cell culture plates (5000 cells/well). Measurement of oxygen consumption rate (OCR) was done in a Seahorse XF96e Extracellular Flux Analyzer (Agilent Technologies) according to the instructions provided by the manufacturer. The measurement was done in XF-DMEM buffer supplemented with 1 mM pyruvate, 2 mM glutamine, and 10 mM glucose. Basal oxygen consumption rate was measured as well as respiration following the addition of 3 μM Oligomycin, 1.5 μM CCCP, and 0.5 μM Antimycin/Rotenone to determine oxygen consumption under differing metabolic conditions.

### 5.12. Gene Ontology, statistical analysis, and presentation of data

Functional and subcellular-component classifications of proteins were extracted from Uniprot [148]. Enrichment and overrepresentation analysis was performed by Panther 18.0 [68]. Statistical analysis was performed using GraphPad Prism 9 (Dotmatics) or Microsoft Excel (Microsoft). Statistical significance was determined using two-tailed T-tests (with Bonferroni correction for multiple comparisons), one-way or two-way ANOVA (with Šidák corrections) as indicated in the respective figure legends. Select figures were generated using art assets from Biorender (biorender.com) with appropriate permissions and licenses (available upon reasonable request).

## Supporting information

S1 Fig Optimization of detergent conditions for cardiac tissue samples. A) Detergent optimization workflow. Enriched membrane fractions were prepared from LV tissues extracted from 21-28wk-old ♂ mice (N = 7). Membrane preparations were pooled, then solubilized using either digitonin (6mg/mg protein), C12E8 (0.5mg/mg protein), Triton X-100 (0.01mg/mg protein) or NP-40 (0.5mg/mg protein)/ Solubilized membranes were fractionated into 35 fractions via BNE and subject to MS-based proteomics analysis. A published R-script (mCP) was used to identify protein complexes within the resultant dataset, using the CORUM v. 4.0 protein complex library as a ground-truth. B) BNE analysis of ventricular membrane preparations solubilized in digitonin, C12E8, Triton X-100 or NP40. Approximate fractions used for complexome-profiling analysis shown to the left. Molecular-weight calibration shown on the far left. C) Number of protein complexes detected following solubilization with the listed detergent. Use of C12E8 resulted in detection of a greater number of complexes. Full list of protein-complex IDs in S2 Fig. Protein complexes detected using mCP coupled to the CORUM protein complex library as described in A).
(TIF)

S2 Fig List of protein complexes detected in cardiac samples solubilized in various detergent conditions. Corresponds to results shown in in S1 Fig.
(PDF)

S3 Fig SEC-MS CP analysis of 28wk-old adult WT and R14^Δ/+^ mice (Cohort #1). A-C) UV-absorption chromatogram (A) and molecular-weight calibration curve (B-C) for SEC-MS experiment shown in Fig 2. D-G) Elution profiles for additional protein complexes located in cytosolic (Coatamer), integral SER membrane (Sec61-translocon), integral plasma-membrane (Cav1.2 and Kir6.2 multimers) and sarcomeric (Titin) compartments. Elution profiles subject to curve smoothing as in Fig 2.
(TIF)

S4 Fig PLN-containing SER Ca^2+^-handling supercomplex is not affected in R14^Δ/+^ mice. A) PLN colocalization with SERCA2a evaluated by STED super-resolution microscopy in 21–28 wk-old ♂ mice (N = 4). Note that these mice are from a separate independent cohort from those used for complexome profiling. B) Fraction of PLN pixels overlapping with SERC2a, and fraction of SERCA2a pixels overlapping with PLN quantified. C) Fraction of PLN pixels within a given distance from nearest SERCA2a signal. D-F) PLN colocalization with Ryr2 evaluated by STED super-resolution microscopy as in panels A-C. Statistical significance determined using unpaired T-test with Bonferroni correction for multiple comparison (B, E) or two-way ANOVA with Šidák correction for multiple comparisons (C, F): ns = no significant difference.
(TIF)

S5 Fig List of CORUM-defined protein complexes detected in Cohort #1 CP dataset. Data was analyzed using the mCP R-script as described in S1 Fig.
(PDF)

S6 Fig Heatmap analysis for technical reproducibility of Cohort #1 CP dataset (28wk-old WT and R14^Δ/+^). A) Proteins used for heatmap analysis. For our study, we used a cutoff of ≤0.6 for Pearson’s coefficient of correlation (blue dotted line), and decrease in CoM above the 95^th^ percentile (red dotted line). See Fig 5A–5E in the main text for details. For heatmap analysis, we also excluded proteins with an increase in CoM above the 95^th^ percentile cutoff (green dotted line). B) Hierarchical cluster analysis of Cohort #1 CP datasets. Hierarchical clustering performed using NOVA v.0.8.0.0 using default parameters.
(TIF)

S7 Fig SEC-MS CP analysis of Cohort #2 (9wk-old juvenile WT and R14^Δ/+^ mice). A-C) UV-absorption chromatogram (A) and molecular-weight calibration curve (B-C) for SEC-MS experiment shown in Fig 9–10.
(TIF)

S8 Fig Heatmap analysis of technical reproducibility within and between Cohorts #1 and #2. A-B) Identification of proteins complexes present in all datasets. The mCP R-script was used to identify protein complexes present within all 4 datasets (WT and R14^Δ/+^, Cohorts 1 and 2). The CORUM 4.0 protein complex library was used for ground-truth. mCP identified 30 protein complexes present in all 4 datasets, representing a total of 125 constituent proteins. Heatmap analysis of proteins within defined protein complexes common across all 4 datasets. Hierarchical cluster analysis performed using NOVA v.0.8.0.0 using default parameters as in S6 Fig.
(TIF)

S9 Fig PLN co-elutes with a subset of ER-mitochondrial tether proteins and TMEM126B. A-C) Elution profiles of PLN (purple) and ER-mitochondrial contact site components VAPA/B, Calnexin (canx), Bcap31 and VDAC1/2/3 from Cohort #1 CP dataset: adult 28wk-old WT (top) and R14^Δ/+^ (*bottom*). D-F) Elution profiles of PLN (purple) and ER-mitochondrial contact site components VAPA/B, Calnexin (canx), Bcap31 and VDAC1/2/3 from Cohort #2 dataset: juvenile 9wk-old WT (top) and R14^Δ/+^ (*bottom*). G, H) Coelution of PLN and TMEm126B in Cohort #1 CP dataset (TMEM126B not detected in Cohort #2 dataset). Co-fractionating peaks indicated with arrow. Elution profiles subject to curve smoothing as in Fig 2.
(TIF)

S1 Table Cohort 1 WT mouse complexome profiling dataset. SEC-MS CP data obtained from a single 28wk-old ♂ WT mouse. Workflow shown in Fig 2B and assay characterization in S3 Fig.
(XLSX)

S2 Table Cohort 1 R14^Δ/+^ mouse complexome profiling dataset. SEC-MS CP data obtained from a single 28wk-old ♂ R14^Δ/+^ mouse. Workflow shown in Fig 2B and assay characterization in S3 Fig.
(XLSX)

S3 Table Annotation of CORUM v4.0 database. CORUM 4.0 database accessed on July 2024. “Cell line” and “GO description” information retrieved from database (Spreadsheet 1). Manual annotation of function (Spreadsheet 2) based on keywords present within “GO description”.
(XLSX)

S4 Table PERCOM analysis on Cohort 1 CP dataset. (XLSX)

S5 Table Cohort 2 WT mouse complexome profiling dataset. SEC-MS CP data obtained from a single “juvenile” 9wk-old ♂ WT mouse. Workflow shown in Fig 10A and assay characterization in S7 Fig.
(XLSX)

S6 Table Cohort 1 WT mouse complexome profiling dataset. SEC-MS CP data obtained from a single “juvenile” 9wk-old ♂ R14^Δ/+^ mouse. Workflow shown in Fig 10A and assay characterization in S7 Fig.
(XLSX)

S1 Text Supporting materials and methods. Corresponds to super-resolution microscopy shown in S4 Fig and hierarchical clustering in S6 and S8 Figs.
(PDF)
