# Supplementary figures and images for: Unbiased complexome profiling and global proteomics analysis reveals mitochondrial impairment and potential changes at the intercalated disk in presymptomatic R14Δ/+ mice hearts
Source: PLoS One. 2024 Oct 24;19(10):e0311203. doi: 10.1371/journal.pone.0311203 (PMC11501035; doi:10.1371/journal.pone.0311203)

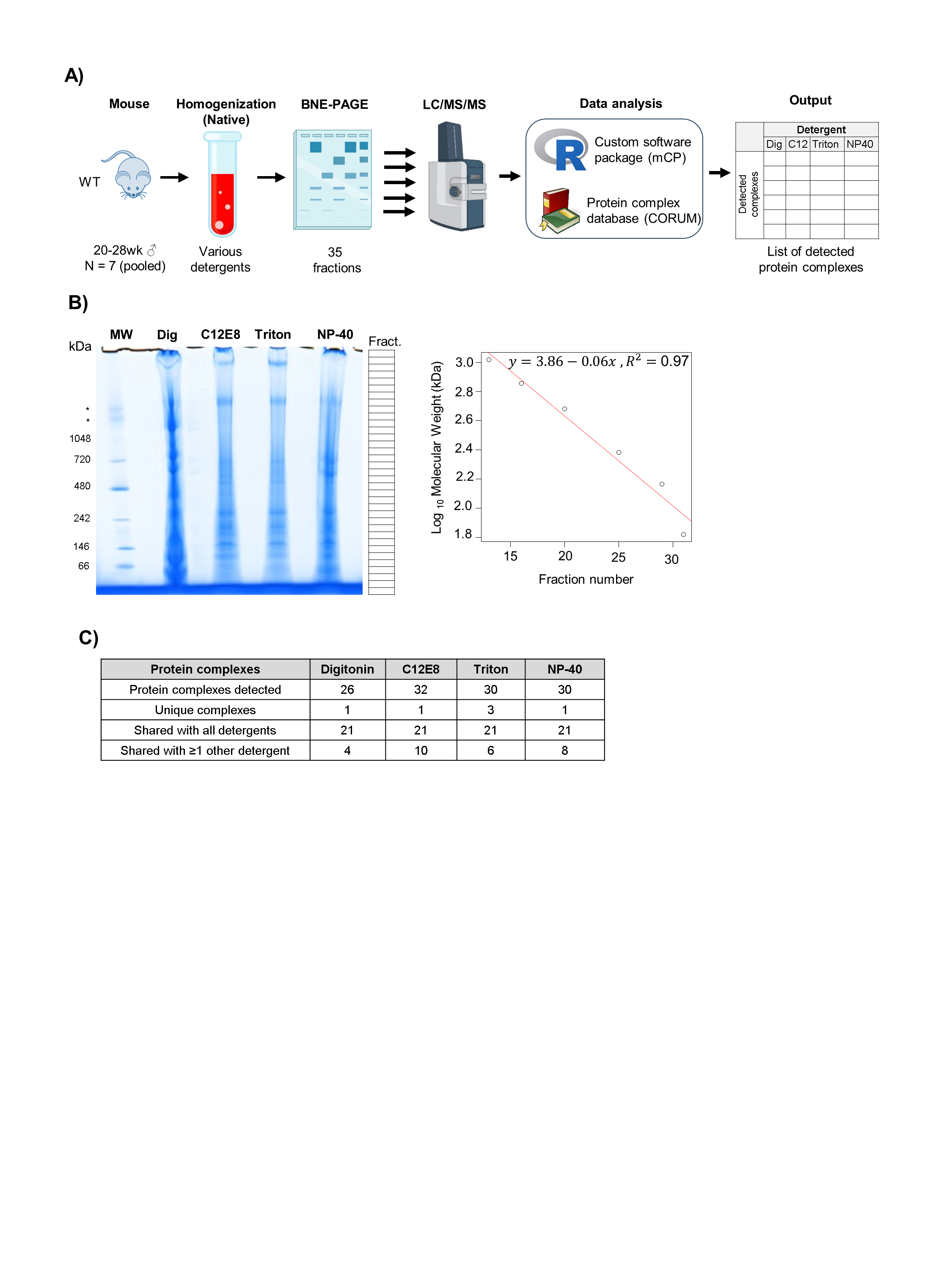

Supplement: S1 Fig — A) Detergent optimization workflow. Enriched membrane fractions were prepared from LV tissues extracted from 21-28wk-old ♂ mice (N = 7). Membrane preparations were pooled, then solubilized using either digitonin (6mg/mg protein), C12E8 (0.5mg/mg protein), Triton X-100 (0.01mg/mg protein) or NP-40 (0.5mg/mg protein)/ Solubilized membranes were fractionated into 35 fractions via BNE and subject to MS-based proteomics analysis. A published R-script (mCP) was used to identify protein complexes within the resultant dataset, using the CORUM v. 4.0 protein complex library as a ground-truth. B) BNE analysis of ventricular membrane preparations solubilized in digitonin, C12E8, Triton X-100 or NP40. Approximate fractions used for complexome-profiling analysis shown to the left. Molecular-weight calibration shown on the far left. C) Number of protein complexes detected following solubilization with the listed detergent. Use of C12E8 resulted in detection of a greater number of complexes. Full list of protein-complex IDs in S2 Fig. Protein complexes detected using mCP coupled to the CORUM protein complex library as described in A). (TIF) [file pone.0311203.s001.TIF]

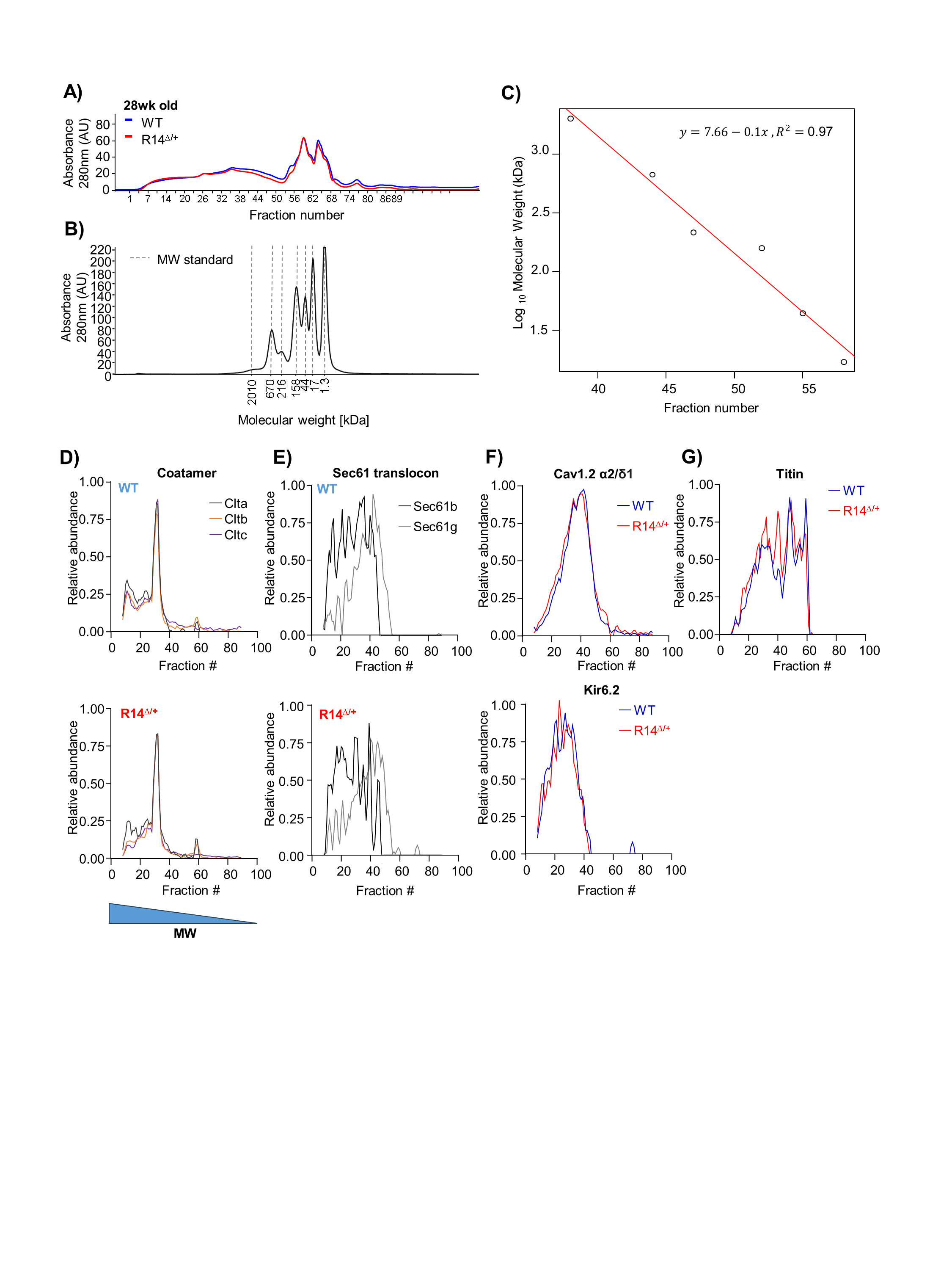

Supplement: S3 Fig — A-C) UV-absorption chromatogram (A) and molecular-weight calibration curve (B-C) for SEC-MS experiment shown in Fig 2. D-G) Elution profiles for additional protein complexes located in cytosolic (Coatamer), integral SER membrane (Sec61-translocon), integral plasma-membrane (Cav1.2 and Kir6.2 multimers) and sarcomeric (Titin) compartments. Elution profiles subject to curve smoothing as in Fig 2. (TIF) [file pone.0311203.s003.TIF]

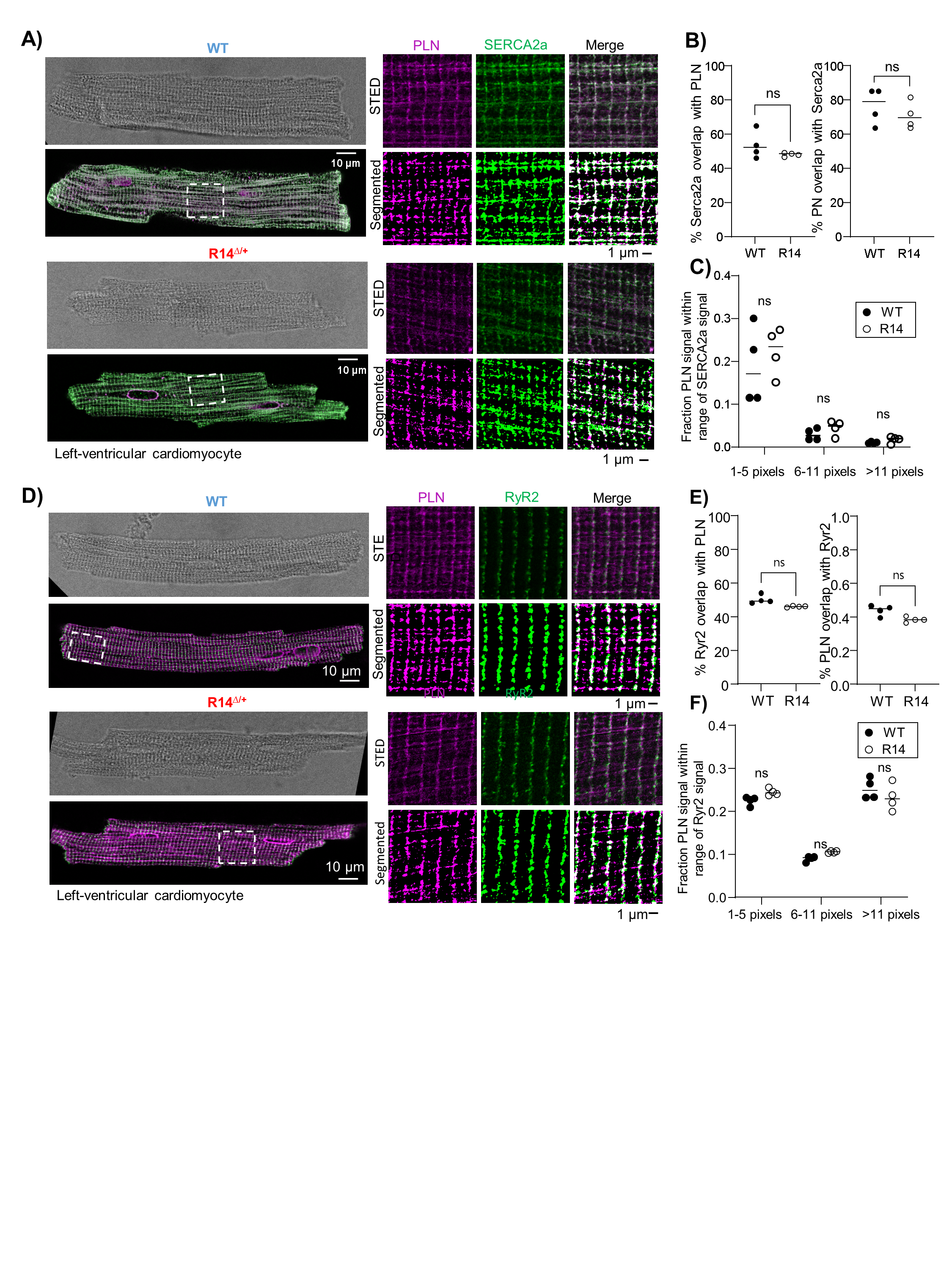

Supplement: S4 Fig — A) PLN colocalization with SERCA2a evaluated by STED super-resolution microscopy in 21–28 wk-old ♂ mice (N = 4). Note that these mice are from a separate independent cohort from those used for complexome profiling. B) Fraction of PLN pixels overlapping with SERC2a, and fraction of SERCA2a pixels overlapping with PLN quantified. C) Fraction of PLN pixels within a given distance from nearest SERCA2a signal. D-F) PLN colocalization with Ryr2 evaluated by STED super-resolution microscopy as in panels A-C. Statistical significance determined using unpaired T-test with Bonferroni correction for multiple comparison (B, E) or two-way ANOVA with Šidák correction for multiple comparisons (C, F): ns = no significant difference. (TIF) [file pone.0311203.s004.TIF]

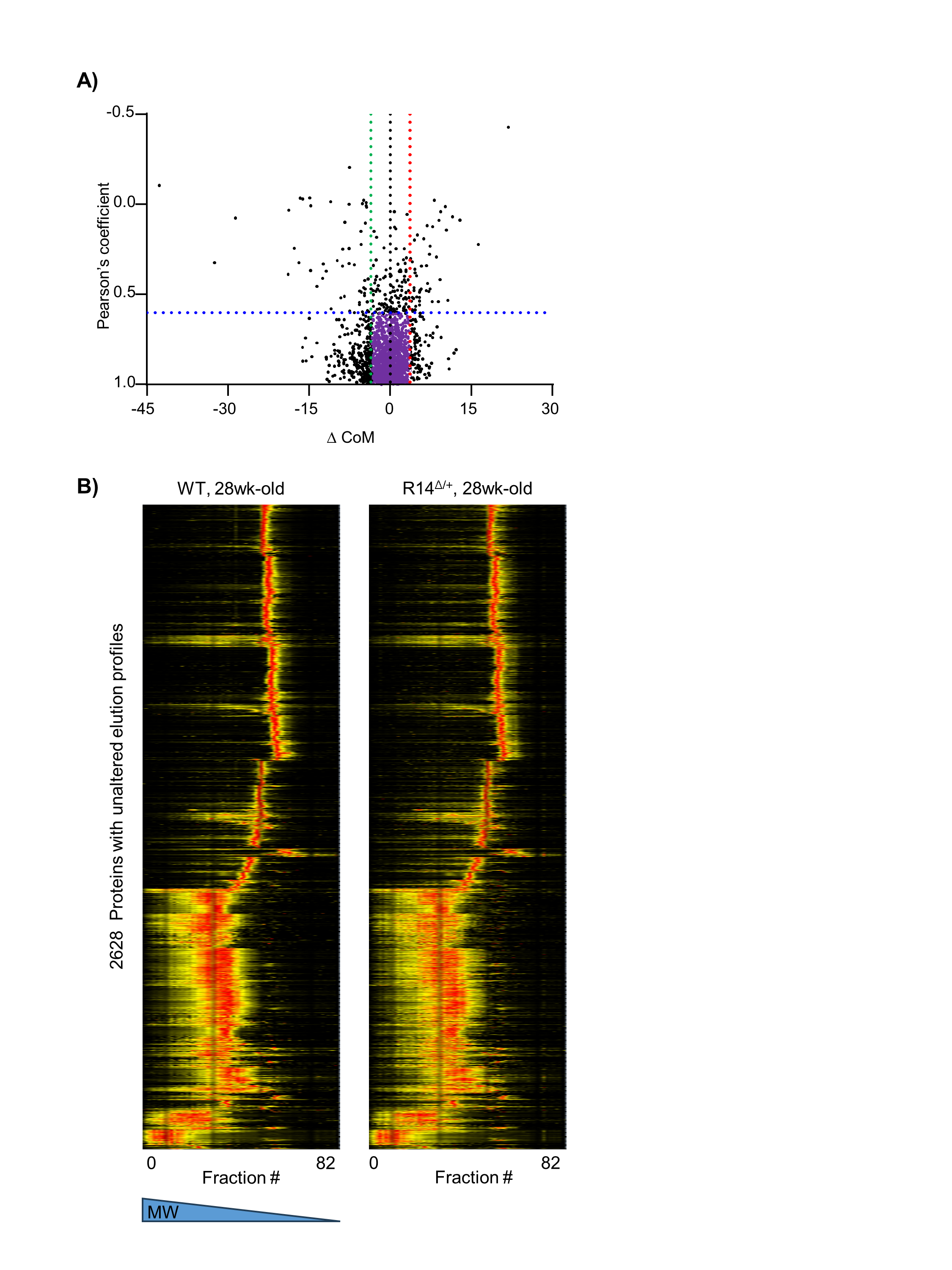

Supplement: S6 Fig — A) Proteins used for heatmap analysis. For our study, we used a cutoff of ≤0.6 for Pearson’s coefficient of correlation (blue dotted line), and decrease in CoM above the 95th percentile (red dotted line). See Fig 5A–5E in the main text for details. For heatmap analysis, we also excluded proteins with an increase in CoM above the 95th percentile cutoff (green dotted line). B) Hierarchical cluster analysis of Cohort #1 CP datasets. Hierarchical clustering performed using NOVA v.0.8.0.0 using default parameters. (TIF) [file pone.0311203.s006.TIF]

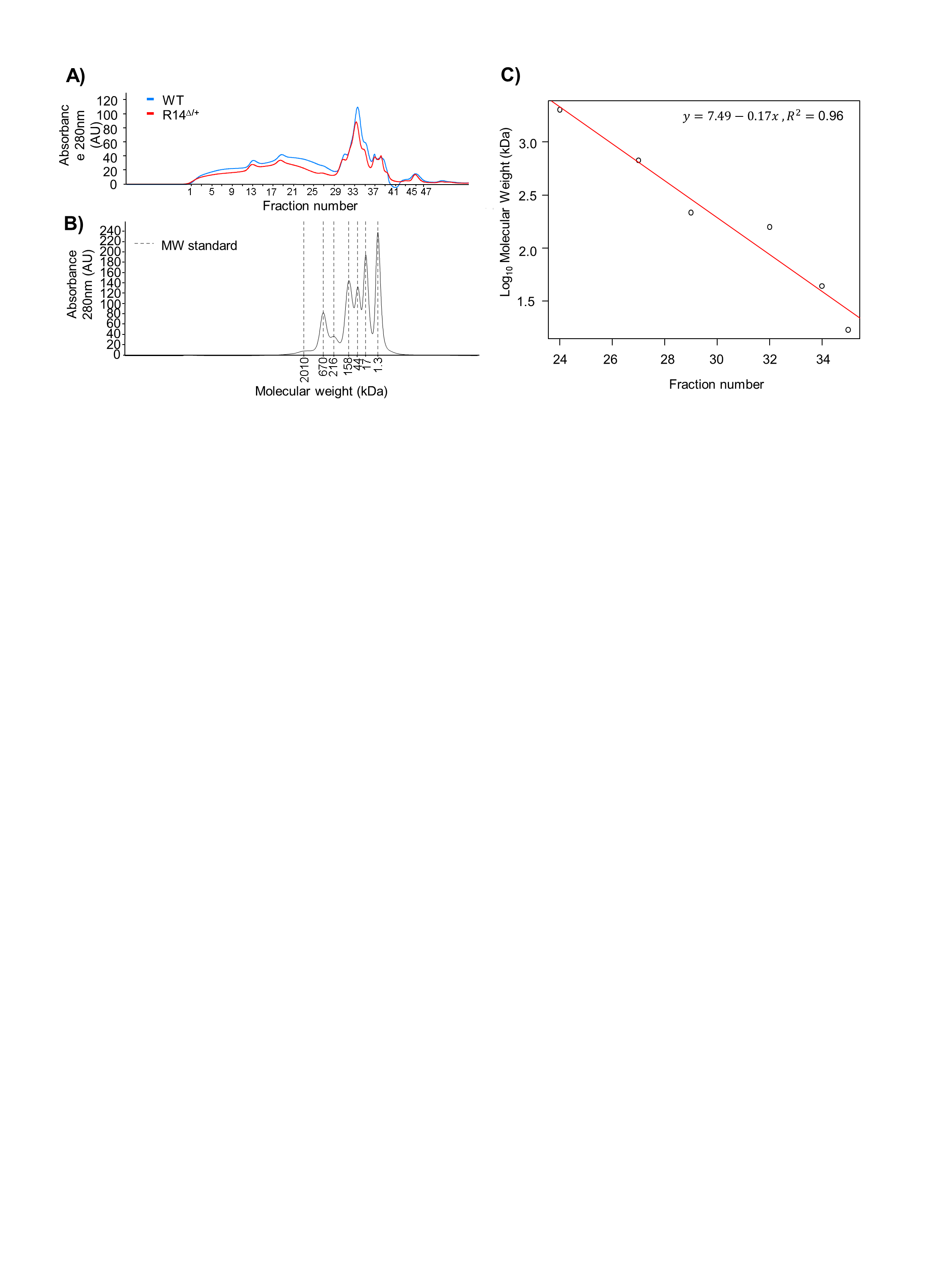

Supplement: S7 Fig — A-C) UV-absorption chromatogram (A) and molecular-weight calibration curve (B-C) for SEC-MS experiment shown in Fig 9–10. (TIF) [file pone.0311203.s007.TIF]

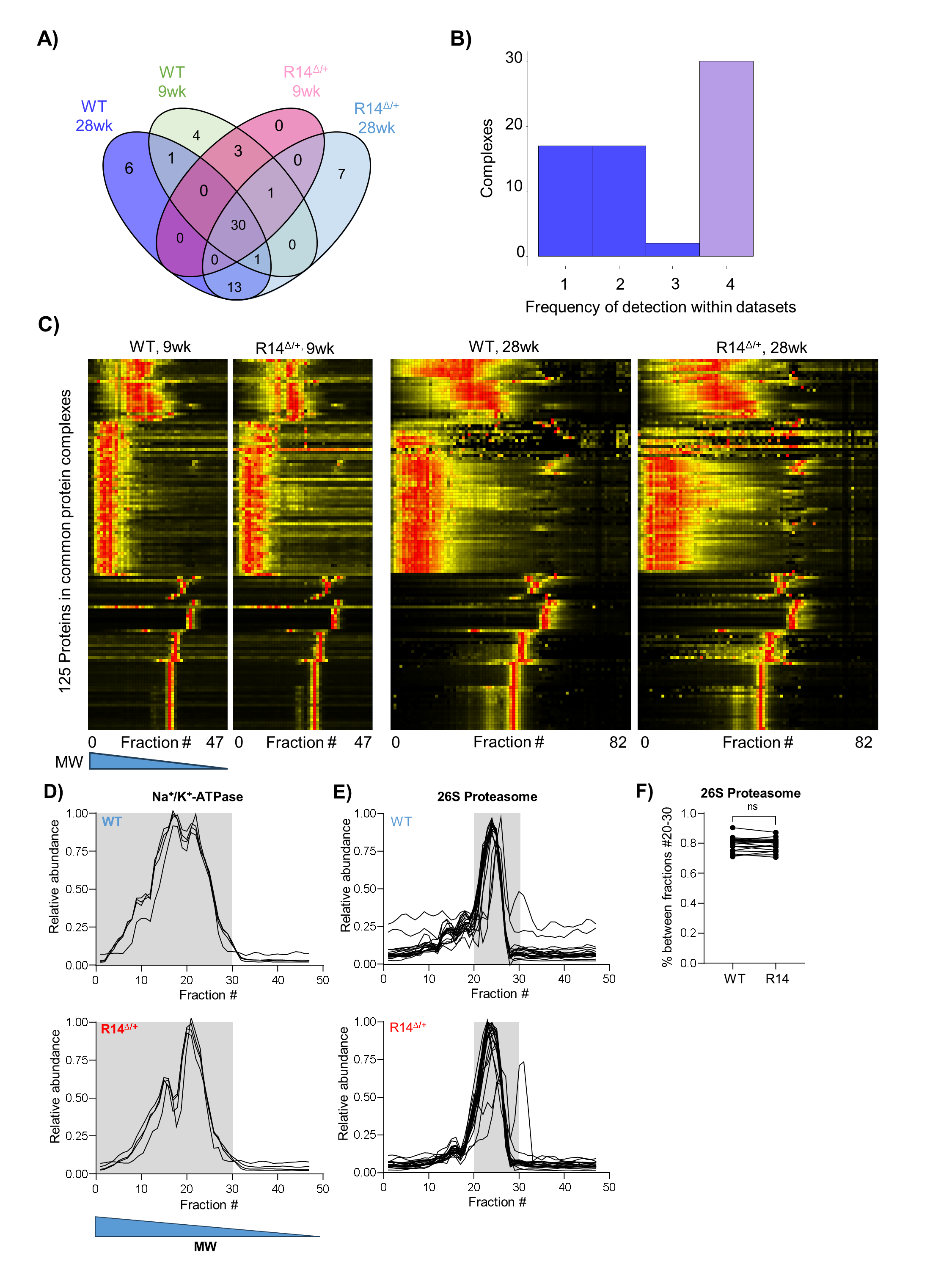

Supplement: S8 Fig — A-B) Identification of proteins complexes present in all datasets. The mCP R-script was used to identify protein complexes present within all 4 datasets (WT and R14Δ/+, Cohorts 1 and 2). The CORUM 4.0 protein complex library was used for ground-truth. mCP identified 30 protein complexes present in all 4 datasets, representing a total of 125 constituent proteins. Heatmap analysis of proteins within defined protein complexes common across all 4 datasets. Hierarchical cluster analysis performed using NOVA v.0.8.0.0 using default parameters as in S6 Fig. (TIF) [file pone.0311203.s008.TIF]

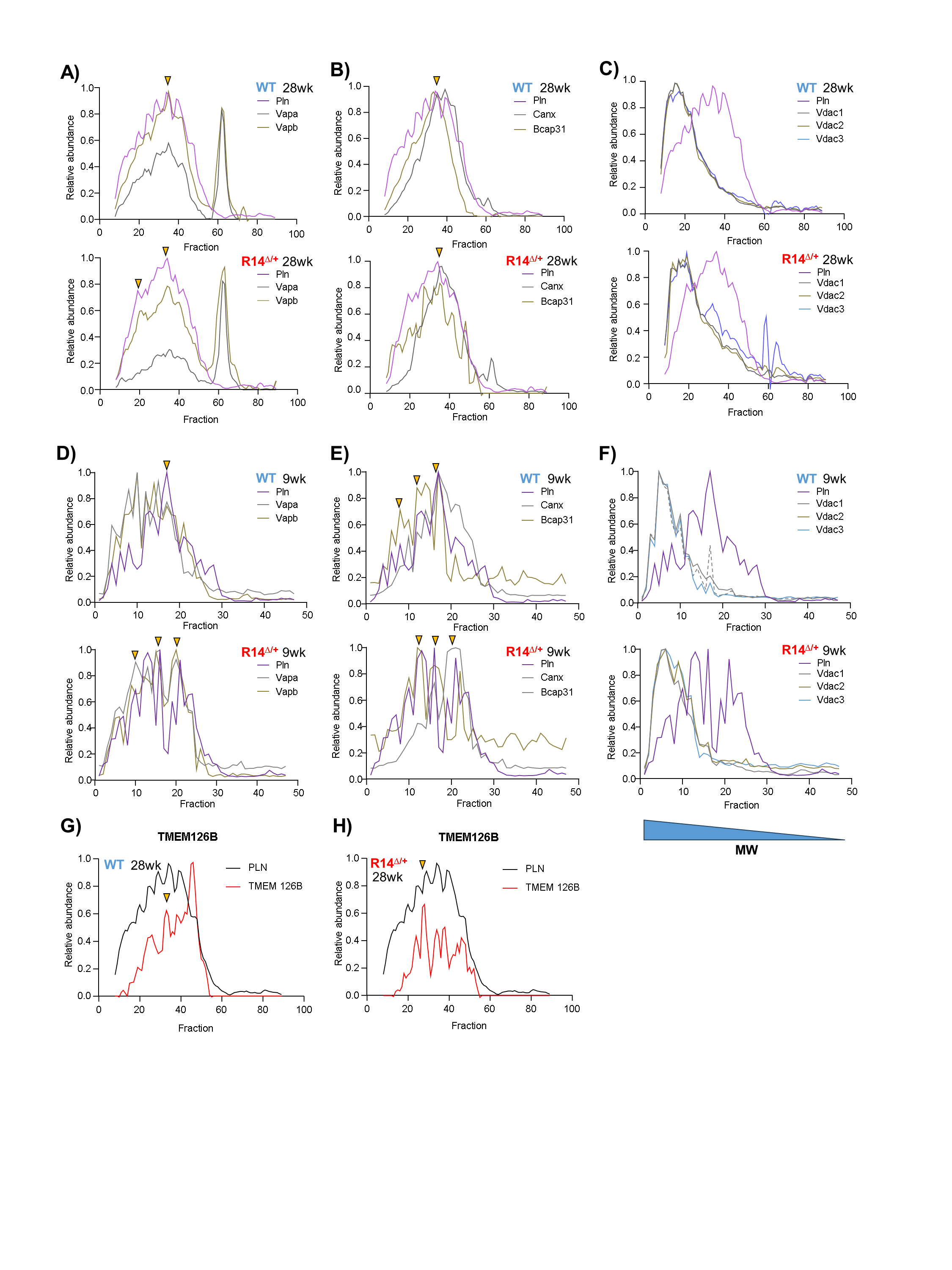

Supplement: S9 Fig — A-C) Elution profiles of PLN (purple) and ER-mitochondrial contact site components VAPA/B, Calnexin (canx), Bcap31 and VDAC1/2/3 from Cohort #1 CP dataset: adult 28wk-old WT (top) and R14Δ/+ (bottom). D-F) Elution profiles of PLN (purple) and ER-mitochondrial contact site components VAPA/B, Calnexin (canx), Bcap31 and VDAC1/2/3 from Cohort #2 dataset: juvenile 9wk-old WT (top) and R14Δ/+ (bottom). G, H) Coelution of PLN and TMEm126B in Cohort #1 CP dataset (TMEM126B not detected in Cohort #2 dataset). Co-fractionating peaks indicated with arrow. Elution profiles subject to curve smoothing as in Fig 2. (TIF) [file pone.0311203.s009.TIF]
