# Supplementary material for: Unbiased complexome profiling and global proteomics analysis reveals mitochondrial impairment and potential changes at the intercalated disk in presymptomatic R14Δ/+ mice hearts
Source: PLoS One. 2024 Oct 24;19(10):e0311203. doi: 10.1371/journal.pone.0311203 (PMC11501035; doi:10.1371/journal.pone.0311203)
Supplement: S2 Fig — Corresponds to results shown in in S1 Fig. (PDF) [file pone.0311203.s002.pdf]

CORUM protein complex detected in C12E8 solubilization	Detected subunits	Total subunits
IDH3G STRING COMPLEX	10	10
Caveolins and others	6	14
20S proteasome	14	14
Immunoproteasome	13	14
COP9 signalosome complex	4	8
CCT complex (chaperonin containing TCP1 complex)_1	8	24
CCT complex (chaperonin containing TCP1 complex)_2	8	24
Skeletal muscle sarcoglycan complex SGC, alpha-beta-gamma-delta	4	12
CCT complex (chaperonin containing TCP1 complex)_3	8	24
Skeletal muscle sarcoglycan complex SGC, beta-gamma-delta-zeta	3	4
Smooth muscle sarcoglycan complex SGC, beta-delta-zeta	2	3
Skeletal muscle sarcoglycan complex SGC, alpha-beta-gamma-delta	4	12
Skeletal muscle sarcoglycan complex SGC, alpha-beta-epsilon-gamma	3	4
Skeletal muscle sarcoglycan complex SGC, alpha-beta-gamma-delta	4	12
Skeletal muscle sarcoglycan complex SGC, epsilon-beta-gamma-delta	3	4
Dystrophin-sarcoglycan-syntrophin complex, skeletal muscle	5	6
Sarcoglycan-sarcospan-dystroglycan complex	5	6
Sarcoglycan-sarcospan-complex SG-SPN	4	5
Sarcoglycan-sarcospan-syntrophin-dystrobrevin complex	4	8
Respiratory chain complex I, mitochondrial	29	77
Respiratory chain complex I, mitochondrial	29	77
(ER)-localized multiprotein complex, Ig heavy chains associated	7	10
Succinate dehydrogenase complex II, mitochondrial	4	4
Cytochrome bc1-complex, mitochondrial	9	10
Cytochrome c oxidase, mitochondrial	11	13
Kif13a-AP1 complex	2	4
Itgav-Itgb3-Gsn complex	3	3
Parvulin-associated pre-rRNP complex	23	62
CCT complex (chaperonin containing TCP1 complex), testis specific	7	8
Vps29-Vps35-Vps26a complex	2	3
Dnajc5-Sgta complex	2	3
Ap1g1-Ap2a2-Dcx complex	2	3
