# Supplementary material for: Unbiased complexome profiling and global proteomics analysis reveals mitochondrial impairment and potential changes at the intercalated disk in presymptomatic R14Δ/+ mice hearts
Source: PLoS One. 2024 Oct 24;19(10):e0311203. doi: 10.1371/journal.pone.0311203 (PMC11501035; doi:10.1371/journal.pone.0311203)
Supplement: S5 Fig — Data was analyzed using the mCP R-script as described in S1 Fig. (PDF) [file pone.0311203.s005.pdf]

CORUM protein complex detected in 28wk-old ♂ WT and R14$^{\Delta/+}$ mice	Detected subunits	Total subunits
IDH3G STRING COMPLEX	6	8
9S-cytosolic aryl hydrocarbon (Ah) receptor non-ligand activated complex	10	10
20S proteasome	3	4
Immunoproteasome	14	14
COP9 signalosome complex	13	14
CCT complex (chaperonin containing TCP1 complex)	6	8
CCT complex (chaperonin containing TCP1 complex)	8	24
Gamma-secretase complex (Aph1a, Psen1, Psenen, Ncstn)	8	24
Skeletal muscle sarcoglycan complex SGC, alpha-beta-gamma-delta	2	8
CCT complex (chaperonin containing TCP1 complex)	4	12
Skeletal muscle sarcoglycan complex SGC, beta-gamma-delta-zeta	8	24
Skeletal muscle sarcoglycan complex SGC, alpha-beta-gamma-delta	3	4
Skeletal muscle sarcoglycan complex SGC, alpha-beta-epsilon-gamma	4	12
Skeletal muscle sarcoglycan complex SGC, alpha-beta-gamma-delta	3	4
Skeletal muscle sarcoglycan complex SGC, epsilon-beta-gamma-delta	4	12
Dystrophin-sarcoglycan-syntrophin complex, skeletal muscle	3	4
Sarcoglycan-sarcospan-dystroglycan complex	5	6
Sarcoglycan-sarcospan-complex SG-SPN	5	6
Sarcoglycan-sarcospan-syntrophin-dystrobrevin complex	4	5
Respiratory chain complex I, mitochondrial	5	8
Respiratory chain complex I, mitochondrial	30	77
Succinyl-CoA synthetase, ADP-forming	30	77
Succinyl-CoA synthetase, GDP-forming	2	2
(ER)-localized multiprotein complex, Ig heavy chains associated	2	2
Succinate dehydrogenase complex II, mitochondrial	8	10
Cytochrome bc1-complex, mitochondrial	4	4
Cytochrome c oxidase, mitochondrial	9	10
Gamma-secretase complex (Aph1a, Ncstn, Psen1, Psenen, Tmp21)	10	13
TRPV5-S100A10-annexin 2 complex	3	5
S100A10-annexin 2 complex	2	3
SNARE complex (Vti1b, Stx6, Stx7)	2	2
SNARE complex (Vti1b, Stx7, Stx8, Vamp8)	2	3
SNARE complex (Stx4, Stx6, Stx7, Vamp3, Vamp7, Vamp8, Vti1b)	3	4
Kif13a-AP1 complex	5	7
ERdj3-BiP complex	3	4
Itgav-Itgb3-Gsn complex	2	2
Itga-Itgb1-Ppap2b complex	2	3
Aph1a-Psen1-Ncstn complex	3	3
Gata1-Fog1-MeCP1 complex	2	3
Nkx3.2-SMAD1-SMAD4-HDAC-Sin3A complex	3	13
Drosha complex	2	7
Parvulin-associated pre-rRNP complex	5	8
CCT complex (chaperonin containing TCP1 complex), testis specific	27	62
G protein complex (Hdac4, Gnb1, Gng2)	7	8
G protein complex (Hdac5, Gnb1, Gng2)	2	3
G protein complex (Btk, Gng2, Gnb1)	2	3
Vps29-Vps35-Vps26a complex	2	3
Gamma-secretase complex (Aph1a, Psen1, Psenen, Ncstn)	2	3
Ksr1-PP2A holoenzyme complex (Ppp2r1a, Ppp2r2b, Ppp2ca), PDGF stimulated	2	8
Ksr1-PP2A core enzyme complex (Ppp2r1a, Ppp2ca), untreated	2	4
Ksr1-PP2A core enzyme complex (Ppp2r1a, Ppp2ca), untreated	2	6
Raf1-PP2A holoenzyme complex (Ppp2r1a, Ppp2r2b, Ppp2ca), PDGF stimulated	2	6
Raf1-PP2A core enzyme complex (Ppp2r1a, Ppp2ca), untreated	3	4
p18-p14-Mp1 complex	3	3
Mp1-p14 scaffolding complex	3	3
Ncstn-Psen1 complex	2	2
Rabep1-Ap1g1-Ap1s1 complex	2	2
