# Supplementary material for: Unbiased complexome profiling and global proteomics analysis reveals mitochondrial impairment and potential changes at the intercalated disk in presymptomatic R14Δ/+ mice hearts
Source: PLoS One. 2024 Oct 24;19(10):e0311203. doi: 10.1371/journal.pone.0311203 (PMC11501035; doi:10.1371/journal.pone.0311203)
Supplement: S1 Text — Corresponds to super-resolution microscopy shown in S4 Fig and hierarchical clustering in S6 and S8 Figs. (PDF) [file pone.0311203.s016.pdf]

Unbiased complexome profiling and global proteomics analysis reveals mitochondrial impairment and potential changes at the intercalated disk in presymptomatic $R14^{\Delta/+}$ mice hearts

Supplemental Methods and Materials

Brian Foo[1,2*¶], Hugo Amedei[3¶], Surmeet Kaur[3,#a], Samir Jaawan[1,2], Angela Boshnakovska[4], Tanja Gall[4], Rudolf A. de Boer[5,6], Herman H.W. Silljé[5], Henning Urlaub[3,7], Peter Rehling[4], Christof Lenz[3,7*], Stephan E. Lehnart[1,2*]

[1] University Medical Center Göttingen, Heart Research Center Göttingen, Cellular Biophysics and Translational Cardiology Section, Göttingen, Germany.

[2] Cluster of Excellence "Multiscale Bioimaging: from Molecular Machines to Networks of Excitable Cells" (MBExC), University of Göttingen, Germany.

[3] University Medical Center Göttingen, Department of Clinical Chemistry, Göttingen, Germany.

[4] University Medical Center Göttingen, Department of Cellular Biochemistry, Göttingen, Germany.

[5] Department of Cardiology, University Medical Center Groningen, University of Groningen, Groningen, the Netherlands.

[6] Erasmus MC, Cardiovascular Institute, Thorax Center, Department of Cardiology, Rotterdam, the Netherlands.

[7] Max Planck Institute for Multidisciplinary Sciences, Bioanalytical Mass Spectrometry Group, Göttingen, Germany.

[#a]Current Address: Institute of Medical Virology, Justus Liebig University, Giessen, Germany.

*Corresponding authors:

Email: brian.foo@med.uni-goettingen.de (BF), christof.lenz@mpinat.mpg.de (CL) and slehnart@med.uni-goettingen.de (SEL)

[¶]These authors contributed equally to this work.

Supplemental Materials and Methods

Super-resolution confocal microscopy. Cardiomyocytes were isolated from mouse left-ventricle as described in the main text and seeded onto fibronectin-coated glass cover slips. PLN, SERCA2a and RyR2 were labelled using the following antibodies: monoclonal mouse α-PLN (Thermo), polyclonal rabbit α-SERCA2a (Badrilla) and rabbit α-RyR2 (Sigma). Secondary antibodies conjugated to STED-compatible fluorophores (STAR 580 or STAR 635P, Abberoir) were used for labelling. Images were acquired using a Leica TCS SP8 STED system using an HC PL APO C2S 100x/1.40 oil immersion lens. Image analysis was performed on FiJi using a custom macro similar to those used in our earlier works[1,2].

Heirarchical Clustering. Heirarchical clustering performed using NOVA v. 0.8.0.0[3]. Clustering was performed using Pearson correlation for distance calculation, default parameters, average linkage, without normalization, without leaf order optimization, and without elimination of empty rows.

Supplemental References

1. Peper J, Kownatzki-Danger D, Weninger G, Seibertz F, Pronto JRD, Sutanto H, et al. Caveolin3 Stabilizes McT1-Mediated Lactate/Proton Transport in Cardiomyocytes. Circ Res. 2021;128: E102–E120. doi:10.1161/CIRCRESAHA.119.316547
2. Alsina KM, Hulsurkar M, Brandenburg S, Kownatzki-Danger D, Lenz C, Urlaub H, et al. Loss of Protein Phosphatase 1 Regulatory Subunit PPP1R3A Promotes Atrial Fibrillation. Circulation. 2019;140: 681–693. doi:10.1161/CIRCULATIONAHA.119.039642
3. Giese H, Ackermann J, Heide H, Bleier L, Dröse S, Wittig I, et al. NOVA: A software to analyze complexome profiling data. Bioinformatics. 2015;31: 440–441. doi:10.1093/bioinformatics/btu623
